# Cdk5 Phosphorylation of ErbB4 is Required for Tangential Migration of Cortical Interneurons

**DOI:** 10.1093/cercor/bht290

**Published:** 2013-10-18

**Authors:** Sonja Rakić, Shigeaki Kanatani, David Hunt, Clare Faux, Anna Cariboni, Francesca Chiara, Shabana Khan, Olivia Wansbury, Beatrice Howard, Kazunori Nakajima, Margareta Nikolić, John G. Parnavelas

**Affiliations:** 1 Department of Cell and Developmental Biology, University College London, London WC1 6BT, UK; 2 Department of Anatomy, Keio University School of Medicine, Tokyo 160-8582, Japan; 3 Breakthrough Breast Cancer Research Centre, Institute of Cancer Research, London SW3 6JB, UK; 4 Department of Cellular and Molecular Neuroscience, Imperial College School of Medicine, London W12 0NN, UK

**Keywords:** cerebral cortex, interneurons, migration, mouse, phosphorylation

## Abstract

Interneuron dysfunction in humans is often associated with neurological and psychiatric disorders, such as epilepsy, schizophrenia, and autism. Some of these disorders are believed to emerge during brain formation, at the time of interneuron specification, migration, and synapse formation. Here, using a mouse model and a host of histological and molecular biological techniques, we report that the signaling molecule cyclin-dependent kinase 5 (Cdk5), and its activator p35, control the tangential migration of interneurons toward and within the cerebral cortex by modulating the critical neurodevelopmental signaling pathway, ErbB4/phosphatidylinositol 3-kinase, that has been repeatedly linked to schizophrenia. This finding identifies Cdk5 as a crucial signaling factor in cortical interneuron development in mammals.

## Introduction

Dysfunction of cerebral cortical interneurons, which may stem from their abnormal development and migration, has been implicated in the etiology of a number of neurological and psychiatric disorders in humans, such as epilepsy, schizophrenia, and autism (Marín 2012). In recent years, genetically manipulated mouse models have been shown to faithfully mimic many of the molecular, biological, and clinical features of human brain pathologies and, also, revealed the source and migratory behavior of different types of neurons. Specifically, it has been found that interneurons of the cerebral cortex (Cx) originate mainly from the subcortical ganglionic eminence (GE) and follow a long journey to their final destinations in the Cx. Initially, interneurons travel in multiple tangential streams, before turning radially to settle in the correct layer. Evidence suggests that their migration relies on the dynamics of the branched leading processes that efficiently modify their orientation in response to extracellular guidance cues (Ang et al. 2003; Métin et al. 2006; Marin et al. 2010). However, little is known about the signaling molecules, linking external guidance cues, and cytoskeletal responses, crucial for proper interneuron migration into the Cx.

Cdk5, a versatile proline-directed serine/threonine kinase, and its main activator p35, are signaling molecules expressed in neurons of the developing Cx (Dhavan and Tsai 2001). p35/Cdk5 kinase is vital for radial glia-guided locomotion and laminar fate of pyramidal neurons by phosphorylating intracellular substrates associated with the cytoskeleton (Govek et al. 2011). Some have suggested that Cdk5 is not involved in interneuron migration, as it does not appear to control their layer acquisition (Gilmore and Herrup 2001; Hammond et al. 2004). However, our earlier work clearly indicated that p35/Cdk5 regulates cortical interneuron directionality and leading process branched morphology, during their tangential phase of migration, in a cell-autonomous manner (Rakić et al. 2009). Here, we aimed to identify Cdk5 substrates solely expressed in cortical interneurons and associated with their migration in order to establish the specific role of p35/Cdk5 in these cells.

ErbB (v-erb-a erythroblastic leukemia viral oncogene homolog) proteins (1–4) are members of the tyrosine protein kinase family and the epidermal growth factor (EGF) receptor subfamily. ErbBs interact with ligands of the EGF superfamily, including neuregulins (NRGs), dimerize, catalytically activate each other by cross-phosphorylation, and then stimulate various signaling pathways. ErbB4, the only ErbB member with multiple isoforms (Junttila et al. 2000), mediates cell migration (Golding et al. 2000; Anton et al. 2004; Sakakibara and Horwitz 2006) via phosphatidylinositol (PI) 3-kinase pathway (Kainulainen et al. 2000; Gambarotta et al. 2004). In the forebrain, ErbB4 expression and function have been constrained to a subpopulation of migrating cortical interneurons (Yau et al. 2003; Flames et al. 2004; Li et al. 2012) that largely differentiate into fast-spiking, parvalbumin (PV)-positive cells (Marín 2012).

Here, we report that p35/Cdk5 directs tangential migration of cortical interneurons by regulating the ErbB4/PI3-kinase signaling pathway at the pallial/subpallial border (PSB). We first showed that the Cyt1 isoform of ErbB4, coupled to PI3-kinase, emerges when interneurons reach the PSB and enter the Cx streaming toward sources of ErbB4 ligands (i.e., NRG1, NRG3, and heparin-binding EGF (HB-EGF)). We then identified ErbB4 as a novel p35/Cdk5 kinase substrate, and demonstrated that Cdk5 is an upstream regulator of ErbB4/PI3-kinase signaling by positively controlling tyrosine phosphorylation of the PI3-kinase docking site Y1056. Further, we found that Cdk5-dependent ErbB4/PI3-kinase signaling cascade regulates interneuron polarity, directionality, and leading process morphology at the PSB. Finally, we showed that lack of Cdk5 activity in *p35* knockout mice (KO) leads to permanent reduction in the final number of a subtype of interneurons (i.e., PV- and somatostatin (SST)-positive) that may affect neuronal circuit formation, thus increasing the risk of neurodevelopmental disorders, such as schizophrenia.

## Materials and Methods

### Mouse Lines

*p35*, *Cdk5*, *GAD67-GFP* (Δneo), ErbB4HER4^heart^, and ErbB4^HET^mice were used in this study (see Supplementary material). All procedures were performed under license, and in accordance to regulations of the UK Home Office, Japan Neuroscience Society and Keio University School of Medicine.

### Fluorescence-Activated Cell Sorting (FACS) of GFP^GAD67(+)^ Cells

GABAergic (GFP-positive) and non-GABAergic (GFP-negative) cells, from Cx and GE of *GAD67-GFP* transgenic mice at E13.5 and E15.5 were isolated by FACS method as described previously (Faux et al. 2010).

### Cell Lines and Transfection

COS7 cells were transfected with expression vectors using Lipofectamine 2000 reagent (Invitrogen) according to manufacturer's protocol, and collected after 48 h.

### RT-PCR, Microarray, Immunohistochemistry, Immunoblotting, and Kinase Assay

Standard techniques were used for these analyses and are described in detail in Supplementary material, as well as the sources of antibodies and reagents.

### Phospho-ErbB4-Thr1152 Antibody

Phosphorylation state-specific polyclonal antibody (ab) that specifically recognizes phosphorylated ErbB4 at Thr1152 was generated and purified by Sigma-Genosys (Haverhill, UK) using a rat peptide sequence CELDEEGYM[pThr]PMHDK conjugated to carrier protein KLH injected as antigen in rabbits.

### Cloning and Site-Directed Mutagenesis

JMa-Cyt1 (referred to as Cyt1) and JMa-Cyt2 (referred to as Cyt2) isoforms of ErbB4, as well as ErbB4ΔICD-JMa (referred to as ErbB4ΔICD), truncated for most of the intracellular domain (ICD), were cloned from a rat adult forebrain cDNA library (see Supplementary material). Cyt1 (accession number AY375306.1) and Cyt2 (accession number AY375307.1), submitted to the GenBank by Gambarotta et al. in 2003, entirely matched the sequences obtained in this study. GST-ErbB4_Ala1143-Tyr1262_, containing T1152 (referred to as GST-T1152), was cloned using Cyt1 as a template. Nrg3 (exons 2, 3, 4+CAG+5), encoding the full EGF-like domain, was cloned from a mouse E12.5 embryo cDNA library.

A single-point mutation in the Cdk5 phosphorylation [T1152 (ACT) to A (GCT)] or PI3-kinase-binding site [Y1056 (TAC) to F (TTC)] of ErbB4 or multiple point mutations within the EGF-like domain of Nrg3 [referred as to Nrg3mut: C1 (TGT) to G (GGT), C2 (TGT) to F (TTT), C6 (TGT) to G (GGT), and conserved R (CGT) before C6 to P (CCT)] were introduced using a standard QuikChange^R^ II XL Site-Directed Mutagenesis Kit (Agilent Technologies; see Supplementary material).

### Expression Vectors

Cyt1, Cyt2, Cyt1-T1152A, Cyt1-Y1056F, Cyt2-T1152A, and ErbB4ΔICD were expressed from the mychis B (-) (Invitrogen) or the pCAG-IRES-EGFP (referred to as pCAG; Kawauchi et al. 2003) vector, GST-T1152 and GST-T1152A from the pGEX-4T2 (GE Healthcare) vector, and Nrg3 and Nrg3mut from the pSeqTag2B (Invitrogen) vector. The pCAG-tdTomato vector was obtained by insertion of tdTomato cDNA from the ptdTomato (Clontech) into the pCAG-MCS2 (Kawauchi et al. 2005) vector.

### In Vitro Migration Assays

Chemotactic assay and focal electroporation of MGE followed by whole telencephalic hemisphere culture were performed as reported previously (Kanatani et al. 2008; Rakić et al. 2009) and are described in detail in Supplementary material.

### Quantification of Cells in Embryonic and Adult Forebrain

The total number of immunolabeled cells was manually counted using the MetaMorph software (Molecular Devices). The surface area of the embryonic MGE or adult somatosensory Cx (including adjacent white matter) was measured with the Image J (NIH) program.

### Statistical Analysis

Data were expressed as mean ± standard error of the mean (SEM) and evaluated for significant differences by means of a 2-tailed Student's *t*-test (Microsoft Excel). Error bars represent SEM.

## Results

### ErbB4, But Not Other ErbBs, is Expressed by Migrating Cortical Interneurons

**Figure 1.**
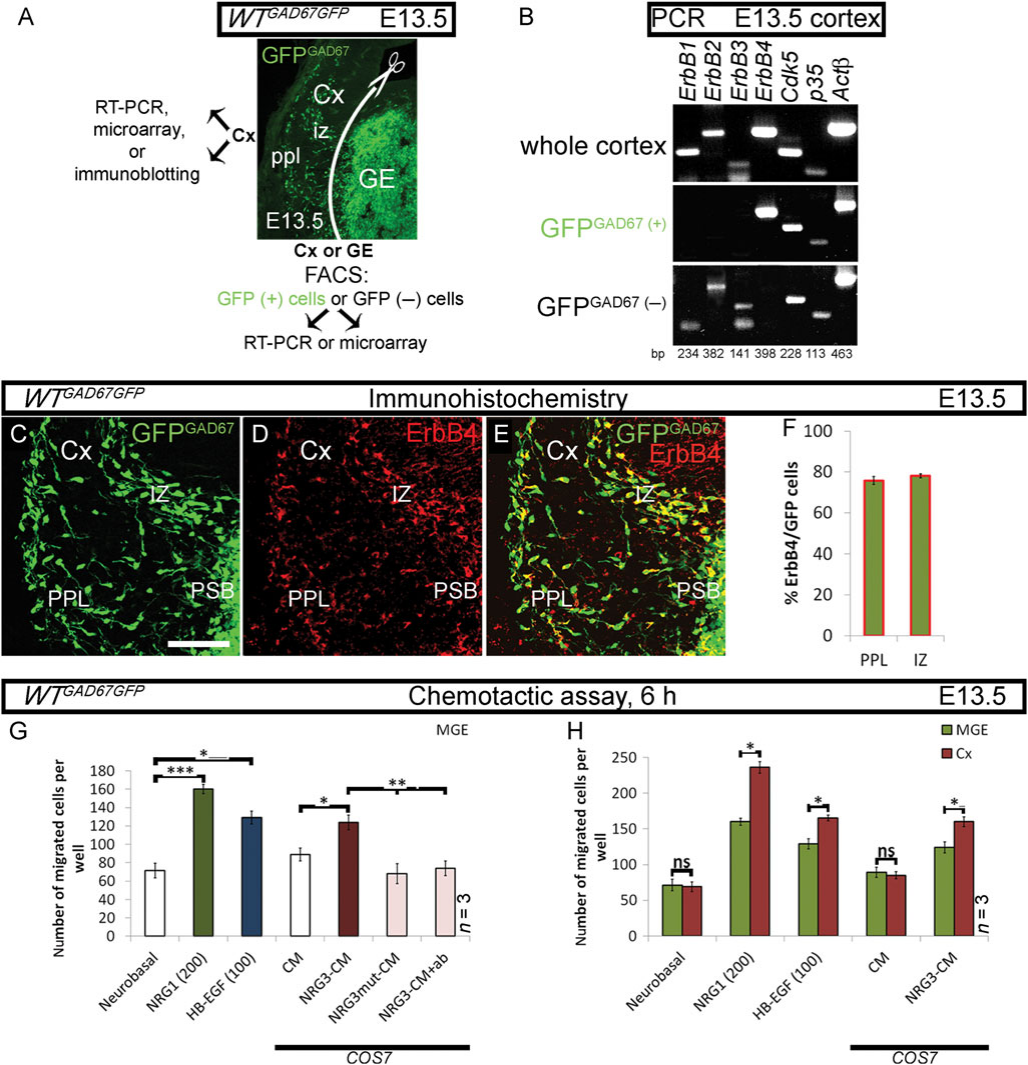
ErbB4 and its ligands facilitate migration of cortical interneurons in vitro*.* (*A*) Forebrain section of a *GAD67GFP* mouse embryo, showing areas/cells used in indicated experiments. GFP^GAD67(+)^ cells represent GABAergic interneurons. (*B*) RT-PCR. *ErbBs*, *p35*, and *Cdk5* expression in the developing Cx. *Actβ* is used as an internal control. (*C*–*F*) Expression of ErbB4 in GFP^GAD67(+)^ cells in the cortical migratory streams. (*G*, *H*) Chemotactic response of MGE- and Cx-derived cells to control (white) or to EGF-like domain of ErbB4 ligands: NRG1β (green), HB-EGF (blue), or NRG3-CM (burgundy). Negative controls for NRG3 (pink): NRG3mut-CM or NRG3-CM treated with a blocking NRG3 ab; (*G*) MGE cells; (*H*) MGE versus Cx cells. *P ≤ 0.05, **P ≤ 0.01, ***P ≤ 0.005, *t*-test. CM, conditioned medium. Bar, 50 μm.

Our aim was to identify substrates of Cdk5 that play a role in the migration of cortical GABAergic interneurons. The differential expression of ErbB growth factor receptors, their ligands, and associated signaling molecules in embryonic (E) forebrain or its GABAergic and non-GABAergic cells, isolated from the *WT^GAD67GFP^* mice by FACS (Fig. 1*A*), was first explored, bearing in mind that some ErbBs are Cdk5 substrates (Fu et al. 2001; Li et al. 2003; Fu et al. 2005), and that ErbB4 is expressed in migrating interneurons (Yau et al. 2003).

End-point PCR, microarray analyses and immunoblotting studies revealed the expression of *ErbB1*, *ErbB2*, and *ErbB4* and very little, if any, *ErbB3* in the Cx (Fig. 1*B*; Supplementary Table 2 and Fig. 1). However, the GFP^GAD67(+)^ cells, isolated from tangential cortical migratory streams at E13.5 and subjected to PCR, expressed ErbB4 and no other members of the ErbB family (Fig. 1*B* and Supplementary Fig. 1). This finding was confirmed in a microarray study targeting all forebrain interneurons, namely, GE and cortical GFP^GAD67(+)^ cells, at E13.5 and E15.5 (Faux et al. 2010, Supplementary Table 2). In addition, immunohistochemistry with ErbB4 ab revealed that about 3 quarters of all GFP^GAD67(+)^ cortical interneurons express ErbB4 in either the preplate (PPL; 77%) or intermediate zone (IZ; 78%) at E13.5 (Fig. 1*C*–*F*). In contrast, the expression of other ErbBs was found in the GFP^GAD67(−)^ cortical cells (Fig. 1*B* and Supplementary Fig. 1). Finally, the expression of *Cdk5* and its activators *p35* and *p39* was also observed in the Cx and in GFP^GAD67(+)^ cells (Fig. 1*B* and Supplementary Table 2).

### Three ErbB4 Ligands Facilitate Cortical Interneuron Migration In Vitro

ErbB4 ligands, namely NRG1, NRG3, and HB-EGF, were abundantly expressed in the noninterneurons (GFP^GAD67(−)^) of the developing forebrain (Supplementary Table 2), and, according to the literature, their topographical distribution was distinct (i.e., cortical plate and proliferative zones of the pallium, PSB and striatum of the subpallium; Supplementary Figs 1 and 2). Therefore, we assessed the effect of these ErbB4 ligands on the migration of interneurons in an in vitro migration assay. Subjects of our analysis were either cells derived from E13.5 medial (M) GE, the site of origin of early-born, ErbB4-expressing interneurons, or Cx. As ErbB3 is hardly expressed in the Cx (Fig. 1*B*; Supplementary Fig. 1 and Table 2) and HB-EGF does not facilitate chemotaxis via ErbB1 (Elenius et al. 1997), only ErbB4 remains as a recognized receptor for the mediation of the chemotactic effects of its ligands at that age (Supplementary Fig. 1). ErbB4 ligands, like other members of the EGF superfamily, contain a common EGF-like domain essential for their activity. This domain has a characteristic structure with 6 cysteine residues that form 3 intramolecular disulfide bonds. Here, we used either an EGF-like domain of NRG1β and HB-EGF, or conditioned medium (CM) of COS7 cells transfected with a plasmid encoding the EGF-like domain of NRG3. Following the 6-h long assay, we noted: first, MGE cells migrated toward NRG1β and HB-EGF in a dose-dependent manner (Fig. 1*G* and Supplementary Fig. 1). Second, MGE cells were attracted to NRG3-CM, which is a significant novel finding, and this action was abolished using either CM of COS7 cells transfected with a plasmid encoding a mutated EGF-like domain of NRG3 (NRG3mut-CM), or NRG3-CM treated with a blocking NRG3 ab that specifically recognizes the EGF-like domain of the ligand (control-CM: 89 ± 7; NRG3-CM: 124 ± 8; NRG3mut-CM: 68 ± 11; NRG3-CM+ab: 74 ± 8; Fig. 1*G*). Finally, the chemotactic response of cortical cells to ErbB4 ligands was peculiarly more robust than that of MGE cells (control: Cx 69 ± 7 vs. MGE 71 ± 8; NRG1β: Cx 236 ± 8 vs. MGE 160 ± 5; HB-EGF: Cx 165 ± 4 vs. MGE 129 ± 7; control-CM: Cx 85 ± 5 vs. MGE 89 ± 7; NRG3-CM: Cx 160 ± 7 vs. MGE 124 ± 8; Fig. 1*H*). In summary, GABAergic cells are attracted by NRG1, NRG3, and HB-EGF in vitro.

### Cortical Interneurons Upregulate *Cyt1* Expression in the Pallium

**Figure 2.**
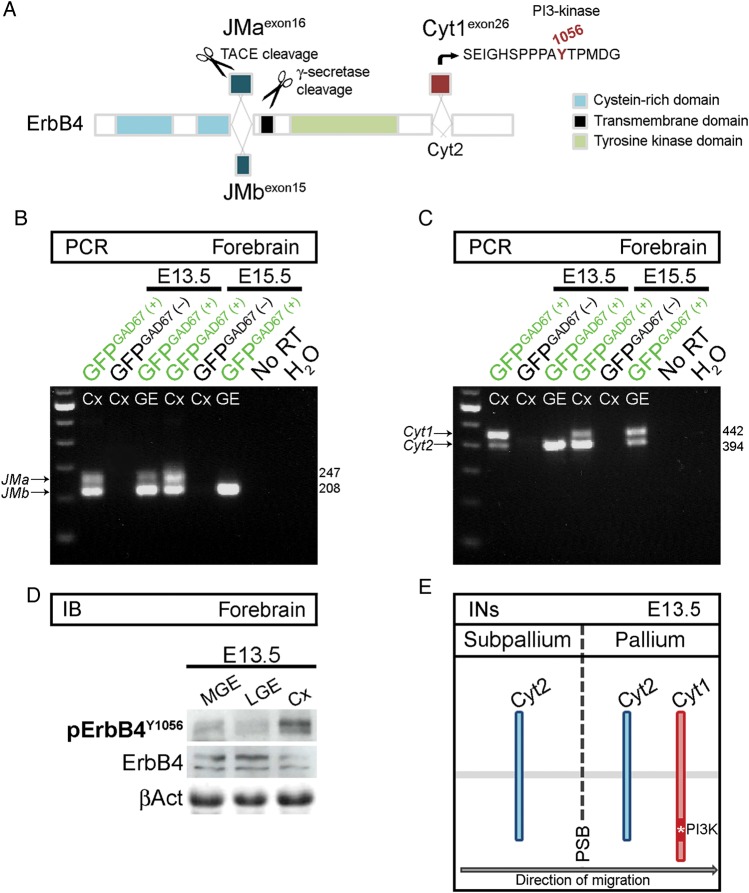
Cortical interneurons upregulate *Cyt1* expression in the pallium. (*A*) Schematic of ErbB4, illustrating domains and isoforms of the receptor (modified from Sundvall et al. 2008). JMa isoform, but not JMb, is susceptible to proteolytic cleavage. Cyt1 isoform contains a unique tyrosine residue (Y1056), absent in Cyt2, which serves as a binding site for PI3-kinase. (*B*, *C*) RT-PCR. *ErbB4* isoform expression in FACS-purified forebrain cells from *GAD67GFP* mice at the indicated time points. GFP^GAD67(+)^ cells represent GABAergic interneurons. (*D*) Immunoblots of protein lysates from the MGE, LGE, and Cx of E13.5 mice, showing phosphorylation (p) of ErbB4 on Y1056. ErbB4 and βAct serve as loading controls. (*E*) Schematic, illustrating upregulation of *Cyt1* as early-born interneurons (INs) depart from the subpallium, cross the PSB and enter the pallium. Cyt, cytoplasmic; JM, juxtamembrane; RT, reverse transcriptase; TACE, tumor necrosis factor-α converting enzyme.

The signaling mechanisms by which ErbB4 exerts its functions in cell migration are only partly understood. Unlike other members of ErbB receptor family, this gene is subject to differential promoter usage and alternative splicing (Junttila et al. 2000; Sundvall et al. 2008). On the one hand, extracellular juxtamembrane (JM) isoforms are either sensitive (JMa) or resistant (JMb) to proteolytic cleavage (Fig. 2*A*). On the other, cytoplasmic isoforms, Cyt1 and Cyt2, differ by the presence (Cyt1) or absence (Cyt2) of a binding site for PI 3-kinase (tyrosine Y1056; Fig. 2*A*); the coupling of Cyt1 with PI3-kinase/Akt pathway stimulates chemotaxis (Kainulainen et al. 2000; Gambarotta et al. 2004). Here, we observed temporal and spatial regulation of ErbB4 isoform expression in forebrain interneurons (GFP^GAD67(+)^; Fig. 2*B*,*C*). Importantly, GFP^GAD67(+)^ cells in the GE, but not in the Cx, at E13.5, lacked expression of the PI3-kinase-binding/chemotaxis-mediating ErbB4 isoform (Cyt1; Fig. 2*C*). Accordingly, by using phosphorylation state-specific ab, we found that ErbB4 was significantly phosphorylated on Cyt1-specific Y1056 in the Cx, but not in the lateral (L) GE or MGE (Fig. 2*D*). Together, these findings suggest that ErbB4/PI3-kinase signaling may be important for migration of interneurons toward and within the Cx (Fig. 2*E*).

### PI3-Kinase and p35/Cdk5 Pathways Regulate ErbB4-Mediated Chemotaxis of Cortical Interneurons In Vitro

**Figure 3.**
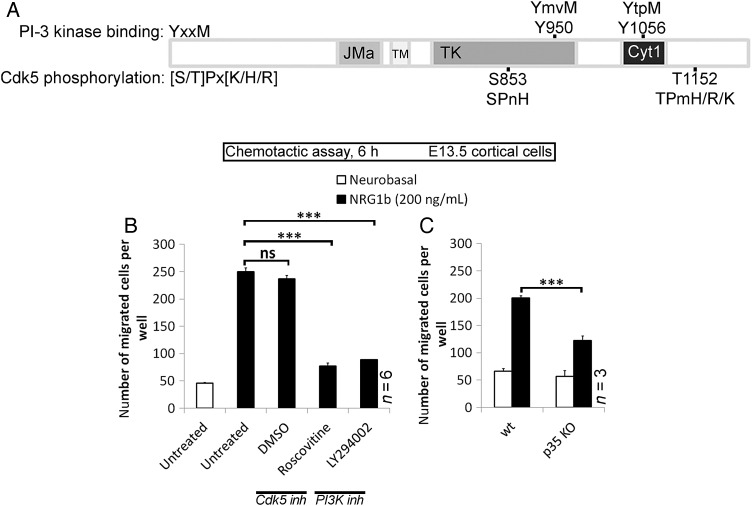
PI3-kinase and p35/Cdk5 pathways regulate ErbB4-mediated chemotaxis of cortical cells in vitro. (*A*) Scansite software, predicting PI3-kinase-binding and Cdk5-targeted phosphorylation sites of ErbB4. (*B*) Chemotactic response of Cx-derived cells, untreated or treated simultaneously with DMSO, Roscovitine (10 μM) or LY294002 (10 μM) for 30 min prior to assay, to NRG1β. (*C*) Chemotactic response of Cx-derived cells, *wt* or *p35*-KO, to NRG1β. *P ≤ 0.05, ***P ≤ 0.005, *t*-test.

Earlier studies have shown the importance of NRG1/ErbB4 (Flames et al. 2004), PI3-kinase (Polleux et al. 2002), and p35/Cdk5 (Rakić et al. 2009) in regulating cortical interneuron migration; however, no relationship between them has yet been described. PI3-kinases, a family of inositol lipid kinase signaling enzymes, are divided into at least 3 distinct classes, I (A and B), II, and III. Developing Cx as well as GAD67^GFP(+)^ interneurons express all but Class IB (Supplementary Table 2). Class IA PI3-kinase (hereafter referred to as PI3-kinase) is the most abundant member of the family in the developing Cx. It consists of a p110 catalytic subunit bound to 1 of 5 regulatory subunits, known as p85s. Recruitment of the p85/p110 complex to receptors occurs via p85 SH2 domains that bind preferentially to phosphorylated tyrosine at the consensus YxxM motif. ErbB4 contains at least 2 YxxM motifs (Y^950^mvM and Y^1056^tpM); the Y^1056^tpM sequence is Cyt1-specific and undoubtedly able to bind/activate PI3-kinase (Fig. 3*A*; Kainulainen et al. 2000). Similarly, there are 2 potential consensus sequences [S/T]Px[K/H/R] for Cdk5 phosphorylation (S^853^PnH and T^1152^TPnH/R) on ErbB4 (Fig. 3*A*); these were identified using the motif search engine Scansite (http://scansite.mit.edu/).

The ability of NRG1β to induce chemotactic response in E13.5 cortical cells with altered either PI3-kinase or Cdk5 signaling was analyzed using an in vitro chemotactic assay. Pretreatment of wild-type (wt) cells with 10 μM LY294002 (PI3-kinase-specific inhibitor) or 10 μM Roscovitine (Cdk5-specific inhibitor), dissolved in dimethyl sulfoxide (DMSO), significantly decreased their migration toward NRG1β compared with untreated or DMSO-treated controls (LY294002: 89 ± 6; Roscovitine: 77 ± 6; untreated: 250 ± 9; DMSO: 237 ± 7; Fig. 3*B*). The role of Cdk5 activity in NRG1β/ErbB4-directed migration was confirmed in E13.5 *p35* litters; the chemotactic response to NRG1β was strong in wt cells (201 ± 10), but significantly reduced in cells derived from *p35 KO* (122 ± 9; Fig. 3*C*). Migration toward the negative control (Neurobasal medium) was weak in both experimental settings (Fig. 3*B*, 46 ± 1; Fig. 3*C*, *wt*: 66 ± 5; *p35 KO*: 57 ± 4). These findings support the notion that the PI3-kinase and Cdk5 signaling pathways regulate NRG1β/ErbB4-dependent cortical interneuron migration in vitro.

### p35/Cdk5 Phosphorylates ErbB4 Receptor on Threonine 1152

**Figure 4.**
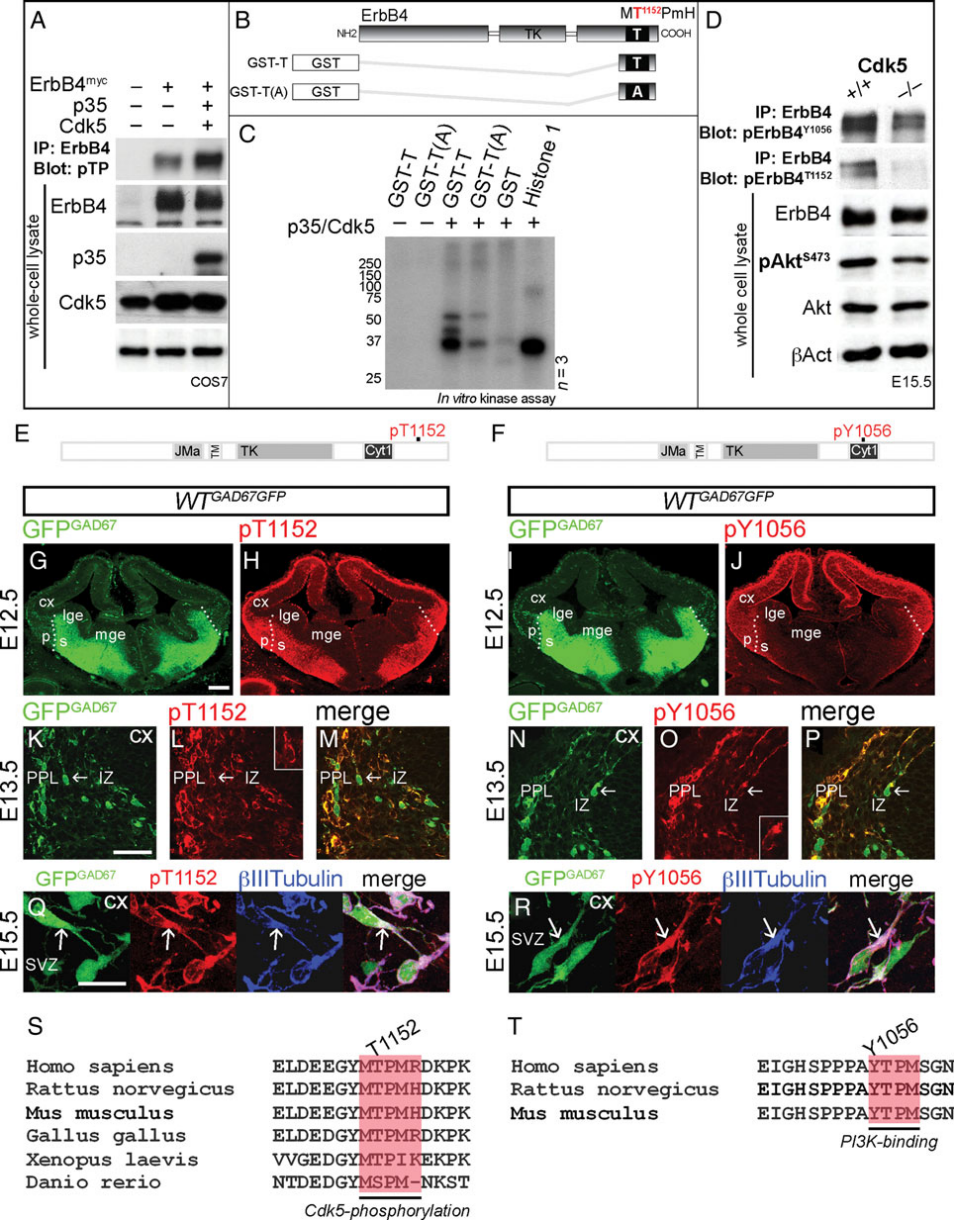
p35/Cdk5 phosphorylation of ErbB4 in vitro and in vivo. (*A*) Immunoprecipitation (IP) of ErbB4^myc^ from COS7 cell protein lysates; COS7 cells were co-transfected with ErbB4^myc^, p35, and Cdk5. Immunoblots of ErbB4 IP protein extracts, revealing p35/Cdk5 phosphorylation of ErbB4 on threonine adjacent to proline (pTP; first and second lanes vs. third lane). Immunoblots of whole-cell protein lysates indicate expression of ErbB4, p35, and Cdk5. βAct serves as a loading control. (*B*) Schematic, illustrating GST-fusion ErbB4 fragments used in a kinase assay. (*C*) Kinase assay, showing phosphorylation of ErbB4 on T1152 by p35/Cdk5. GST and histone 1 serve as negative and positive controls, respectively. (*D*) IP of ErbB4. Immunoblots of ErbB4 IP protein extracts and whole-cell protein lysates from the Cx of E15.5 mice, revealing decreased phosphorylation of ErbB4^T1152^, ErbB4^Y1056^, and Akt^S473^ in the *Cdk5* KOs compared with littermate controls. ErbB4, Akt, and βAct serve as loading controls. (*E*, *F*) Schematic diagrams of ErbB4, showing Cdk5-targeted (T1152; *E*) and PI3-kinase-binding (Y1056; *F*) phosphorylation sites recognized by pErbB4^T1152^ and pErbB4^Y1056^ antibodies, respectively. (*G*–*R*) Forebrain sections of a *GAD67GFP* mouse embryo, at the indicated time points, immunostained for pErbB4^T1152^ and pErbB4^Y1056^ (red). GFP^GAD67(+)^ cells (green) represent GABAergic interneurons. (*G*–*J*) Dotted lines indicate the border between the pallium (p) and the subpallium (s). (*K*–*P*) show pErbB4^T1152^ and pErbB4^Y1056^ in a subset of cortical interneurons (arrows). Higher magnifications are shown in the insets. (*Q*) and (*R*) show that pErbB4^T1152^ and pErbB4^Y1056^ co-localize with β-III-tubulin (blue) in the proximal leading process (arrows) of cortical interneurons. (*S*,*T*) Cdk5-phosphorylation and PI3K-binding sites in ErbB4 are well conserved across species. Bars, 200 μm (*F*–*I*), 50 μm (*J*–*O*), 20 μm (*P*,*Q*). A, alanine; GST, glutathione S-transferase; SVZ, subventricular zone; Y, tyrosine.

A number of studies have suggested a strong correlation between Cdk5 activity and the ErbB receptor pathway (Fu et al. 2001, 2005; Li et al. 2003; Xie et al. 2004, 2007). To determine whether p35/Cdk5 phosphorylates ErbB4 in vitro, we carried out experiments using COS7 cells that naturally express Cdk5, but not p35 or ErbB4. Cdk5 phosphorylates proteins containing a [S/T]Px[K/H/R] site (Fig. 3*A*). We focused on threonine 1152 (T^1152^PmH/R) that lies in close proximity to Cyt1-specific PI3-kinase-binding site Y1056. To initially test whether ErbB4 is a substrate of Cdk5, we used a phosphospecific ab that recognizes a minimal Cdk consensus site, phosphorylated threonine adjacent to a proline (T(PO4)P), and discovered a stronger ErbB4 phosphorylation in COS7 cells that co-expressed p35/Cdk5 compared with other experimental conditions (Fig. 4*A*). To further confirm that ErbB4 is indeed phosphorylated by Cdk5, we performed an in vitro p35/Cdk5 kinase assay using recombinant GST-fused ErbB4 fragments containing either the native T1152 residue (GST-T) or an alanine point mutation (T1152A), which was obtained by site-directed mutagenesis and cannot be phosphorylated [GST-T(A); Fig. 4*B*]. GST and histone 1, an established substrate of Cdk5, were used as negative and positive controls, respectively. This assay revealed a robust phosphorylation by p35/Cdk5 of intact (GST-T), but not mutated [GST-T(A)] ErbB4 fragments (Fig. 4*C*), identifying ErbB4 as a Cdk5 substrate in vitro.

### Cdk5 Promotes ErbB4/PI3-Kinase Activity In Vivo

Next, we hypothesized that Cdk5, by phosphorylating ErbB4 at T1152 in close proximity to PI3-kinase-binding site (Y1056), could regulate ErbB4/PI3-kinase activity. To explore this premise, we looked at the levels of ErbB4 phosphorylation on residue Y1056, as well as amounts of phosphorylated Akt (S473), a downstream molecule in ErbB4/PI3-kinase signaling cascade, in protein lysates of E15.5 forebrain derived from *Cdk5* litters. Decreased phosphorylation of ErbB4^Y1056^ and Akt^S473^ in *Cdk5* KO animals compared with controls, confirmed our hypothesis that phosphorylation of ErbB4 by Cdk5 acts as a positive regulator of ErbB4/PI3-kinase/Akt pathway in the Cx in vivo (Fig. 4*D*).

### Phosphorylated ErbB4^Y1056^ and ErbB4^T1152^ are Expressed in Cortical Interneurons

To evaluate the state of phosphorylation of ErbB4 in cortical interneurons, we used phosphorylation-specific antibodies that recognize Cdk5 consensus site (pErbB4^T1152^; Fig. 4*E*) or Cyt1-specific PI3-kinase-binding site (pErbB4^Y1056^; Fig. 4*F*). To test ab specificity, we co-transfected COS7 cells with plasmids encoding either 1) ErbB4 (Cyt1), ErbB4ΔICD or mutated, phosphorylation-resistant ErbB4^Y1056F^ (pErbB4^Y1056^ ab) or 2) p35/Cdk5 in addition to ErbB4 (Cyt1), ErbB4ΔICD or mutated, phosphorylation-resistant ErbB4^T1152A^ (pErbB4^T1152^ ab), and, after 48-h incubation, treated the cells with 50 ng/mL of NRG1β for 10 min. Immunoblotting of COS7 cells expressing intact or ICD-lacking ErbB4 showed positive or negative signal, respectively, for both phosphorylation-specific antibodies (Supplementary Fig. 3). However, while pErbB4^T1152^ ab did not recognize mutated ErbB4^T1152A^ (Supplementary Fig. 3), there was a reduced signal in ErbB4^Y1056F^-expressing COS7 cells probed with pErbB4^Y1056^ ab compared with positive control (ErbB4), indicating that this ab could identify other possible PI3-kinase-binding consensus sites on the receptor (Chuu et al. 2008, Supplementary Fig. 3). In developing forebrain, both phosphorylated forms of ErbB4 were observed during the peak period of tangential interneuron migration (Fig. 4*G*–*R*). Notably, pErbB4^T1152^ was detected in postmitotic areas of both the subpallium and Cx (Fig. 4*H*), while pErbB4^Y1056^ was prominently observed only at the PSB and in the Cx (Figs 4*J* and 2*C*,*D*). Importantly, immunohistochemical analysis revealed the presence of both pErbB4^T1152^ and pErbB4^Y1056^ in approximately two-thirds of embryonic GFP^GAD67(+)^ cells (Fig. 4*K*–*P* and Supplementary Fig. 3). In addition, pErbB4^T1152^ was detected in adult PV-positive interneurons (Supplementary Fig. 3). At subcellular level, phosphorylated ErbB4 proteins were found to co-localize with β-III-tubulin, a structural component of microtubules, particularly in the initial segment of the interneuron leading process (Fig. 4*Q*,*R*). The fact that the residues that characterize the consensus site for Cdk5 phosphorylation or PI3-kinase-binding of ErbB4 have been conserved across species (Fig. 4*S*,*T*), and are in a state of phosphorylation in migrating interneurons, indicate that they might have an important role in directing these cells from the subpallium towards the cerebral Cx.

### ErbB4 Regulates Cortical Interneuron Directionality and Morphology via Cdk5 and PI3-Kinase Pathways

**Figure 5.**
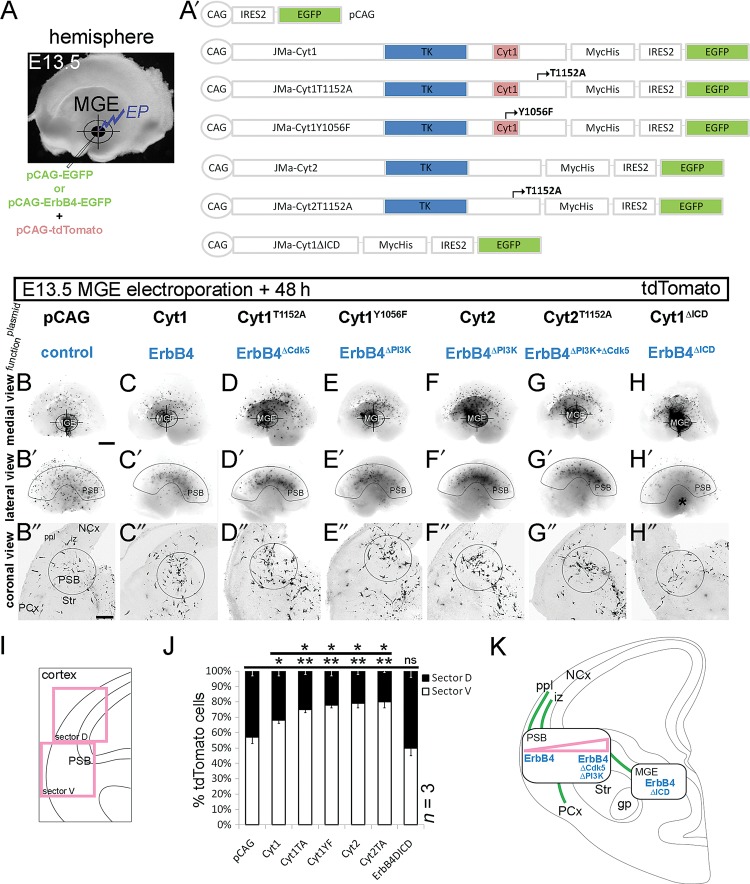
Different ErbB4 signaling pathways regulate tangential distribution of MGE cells. (*A*) Focal electroporation (EP) of control (pCAG) vector and ErbB4 constructs (expressed from pCAG; *A*′), mixed with a CAG-driven tdTomato vector, into the E13.5 MGE of the whole-mouse telencephalic hemisphere. (*B*–*H*″) Fluorescent images of tdTomato, converted to grayscale mode, 48-h post electroporation. (*B*–*H*) Medial view of the telencephalic hemisphere. Target symbol specifies the site of electroporation (MGE). (*B*′–*H*′) Lateral view of the telencephalic hemisphere, depicting the extent of tangential cell spread. The arcuate-shaped outline indicates the position of the PSB. (*H*′) Asterisk, MGE. (*B*″–*H*″) Coronal view of the sectioned telencephalic hemisphere, showing the cortical migratory streams. A circle outlines the PSB. (*I*, *J*) Quantification of the distribution of MGE cells in the Cx. (*I*) Schematic, showing the Cx divided into 2 equal sectors, ventral (marked “V,” which includes the PSB) and dorsal (marked “D”). (*J*) A graph, showing the result of quantification. *P ≤ 0.05, **P ≤ 0.01, *t*-test comparing with pCAG. (*K*) Schematic representations of EP experiments: green lines depict the migratory routes of interneurons, rectangles indicate the sites of abnormal accumulation of cells overexpressing ErbB4 plasmids (blue), and triangle (pink) signifies the severity of the defect. gp, globus pallidus; NCx, neocortex; PCx, paleocortex; Str, striatum. Bar, 250 μm.

To test the function of phosphorylated ErbB4 in tangential migration of cortical interneurons, we performed focal electroporation of MGE of the whole telencephalic hemisphere at E13.5 (Fig. 5*A*), utilizing an array of different ErbB4 constructs encoding the full-length (Cyt1) or genetically modified ErbB4, with the aim of altering Cdk5 phosphorylation (Cyt1^T1152A^), PI3-kinase-binding (Cyt1^Y1056F^ and Cyt2), both Cdk5 and PI3-kinase pathways (Cyt2^T1152A^) or entire ErbB4 intracellular signaling (Cyt1^ΔICD^; Fig. 5*A*′). As a control, we used CAG-driven EGFP vector (pCAG) that also served as a backbone for all ErbB4 constructs (Fig. 5*A*′). To enhance the fluorescent signal, all plasmids were co-transfected with the CAG-driven tdTomato vector; the efficiency of the co-transfection, measured as a ratio between GFP^(+)^ and tdTomato^(+)^ cells, was close to 1 (Supplementary Fig. 4). For all analyses, images of red tdTomato fluorescence, converted to grayscale mode, were used.

The positions of MGE cells were assessed in whole hemispheres and in forebrain sections 48-h postelectroporation. While the medial view of the hemispheres revealed the site of plasmid injection (MGE) (Fig. 5*B*–*H*), the lateral view allowed us to observe the tangential spread of MGE cells in the entire hemisphere (Fig. 5*B*′–*H*′). Images of forebrain sections, after cutting the hemispheres coronally (Fig. 5*B*″–*H*″), depicted the MGE cell migratory streams after passing the LGE; one running toward the piriform cortex (PCx) and the other in the direction of the neocortex (NCx; Fig. 5*B*″,*K*). The latter bifurcated at the level of the PSB and gave rise to 2 streams running through the PPL and IZ (Fig. 5*B*″,*K*). We observed a significant departure from the typical direction of migration in all experimental conditions, except control (pCAG). Strikingly, overexpression of intact ErbB4 (Cyt1) or ErbB4 modified to specifically suppress Cdk5, PI3-kinase or both pathways caused a pronounced phenotype characterized by abnormal accumulation of MGE cells in the PSB (Fig. 5*C*″–*G*″), and revealing an arcuate distribution when the hemisphere was viewed laterally (Fig. 5*C*′–*G*′). These MGE cells, on a coronal view and using a sector analysis (Fig. 5*I*), were largely gathered in the ventral (V) Cx (including the PSB) with fewer cells reaching the dorsal (D) Cx (Cyt1; V: 68 ± 2%; D: 32 ± 3%, 15 sections; Cyt1^T1152A^; V: 75 ± 2%; D: 25 ± 2%, 21 sections; Cyt1^Y1056F^; V: 78 ± 2%; D: 22 ± 3%, 18 sections; Cyt2; V: 79 ± 3%; D: 21 ± 2%, 19 sections; Cyt2^T1152A^; V: 80 ± 4%; D: 20 ± 1%, 13 sections; Fig. 5*J*,*K*) compared with controls (pCAG; V: 57 ± 4%; D: 43 ± 3%, 23 sections; Fig. 5*B*′,*B*″,*J*,*K*). It is worth noting that MGE cells overexpressing the intact ErbB4 receptor (Cyt1) exhibited a milder directionality defect compared with those which carried genetically modified ErbB4 (Fig. 5*C*′,*C*″,*J*,*K*). A complete removal of the ICD of ErbB4 (ErbB4ΔICD) in MGE cells led to a distinct phenotype in which numerous ErbB4ΔICD-expressing cells failed to advance from the MGE toward the LGE and Cx (Fig. 5*H*′,*H*″,*K*); those that successfully migrated across the subpallium and into the Cx, were most likely a subpopulation that did not express ErbB4 in the first place (Fig. 1*C*–*F*).

**Figure 6.**
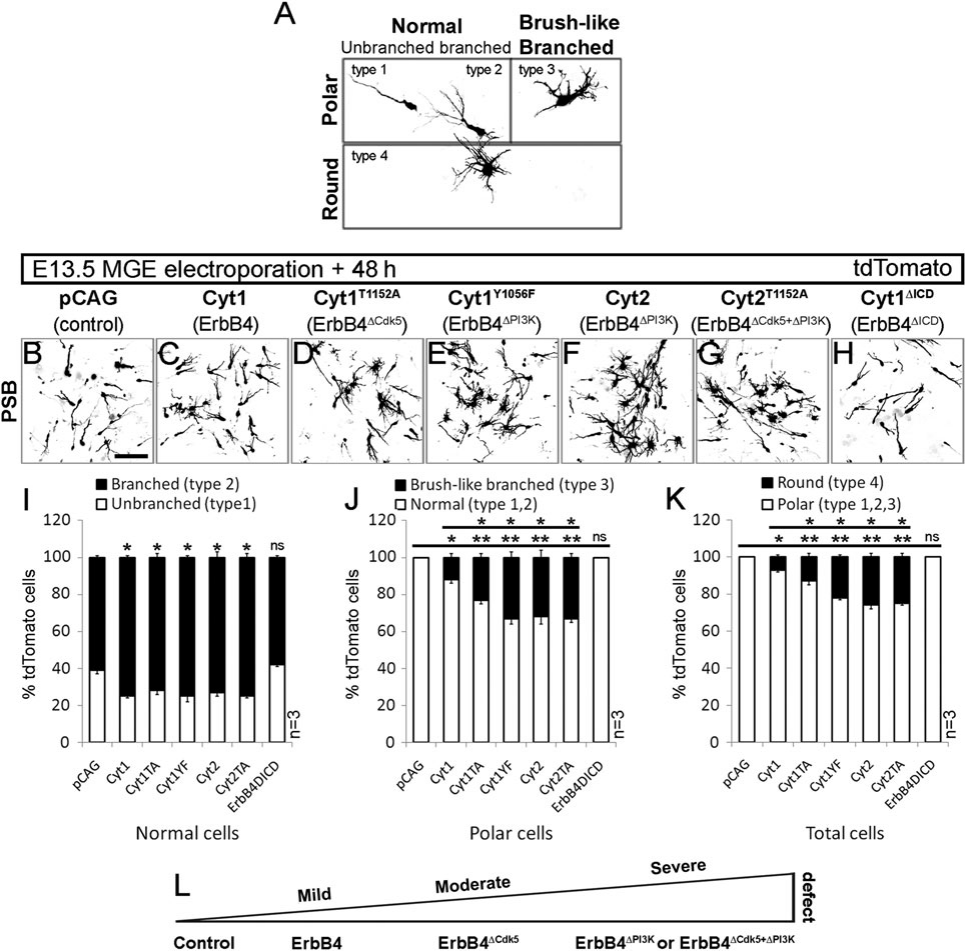
Leading process morphology defects of cortical interneurons after loss of Cdk5 and PI3-kinase ErbB4 signaling. (*A*–*H*) tdTomato fluorescence, converted to grayscale, revealing morphology of migrating interneurons, 48-h postelectroporation of E13.5 MGE with control (pCAG; *B*) and ErbB4 (*C*–*H*) constructs (see Fig. 5). (*A*) Four types of MGE cell-leading process morphologies, observed at the PSB. (*I*) Percentage of unbranched (type 1) and branched (type 2) cells relative to total number of normal (type 1, 2) cells. (*J*) Percentage of brush-like branched (type 3) and normal (type 1, 2) cells relative to total number of polar (type 1, 2, and 3) cells. (*K*) Percentage of round (type 4) and polar (type 1, 2, and 3) cells relative to total cell number (type 1, 2, 3, and 4). (*L*) Schematic, indicating the severity of morphology defect in MGE cells in respect to altered ErbB4 signaling pathway. *P ≤ 0.05 (mild), **P ≤ 0.01 (moderate), ***P ≤ 0.005 (severe), *t*-test comparing against either control or Cyt1. Bar, 50 μm.

We found that genetic manipulation of the ErbB4 signaling pathway in migrating MGE cells not only affected the direction of movement, but also altered their morphologies. For morphometric analysis, we focused on MGE cells situated at the PSB and Cx, and observed 4 types of morphologies (Fig. 6*A*). These appearances were perfectly in keeping with the descriptions reported by Baudoin et al. (2008), who treated MGE cells with nocodazole, a microtubule depolymerizing agent; however, we adopted a slightly different descriptive terminology. Most of the MGE cells exhibited typical polar morphology, with either unbranched/non-bifurcated (type 1; Fig. 6*A*) or branched/bifurcated (type 2; Fig. 6*A*) leading process. In addition, we found polar MGE cells that had a short and thick leading process with numerous protrusions emanating from the main neurite; they had a brushy appearance, hence, we termed them brush-like branched cells (type 3; Fig. 6*A*; Baudoin et al. 2008). Polar MGE cells, normal or brush-like branched, were present in both PSB and Cx. Finally, we observed round cells that showed no polarity, and had numerous extensions without forming a leading process (type 4; Fig. 6*A*); these were found in the trajectory between MGE and Cx, with the maximum accumulation at the PSB.

Alterations in the morphology of MGE cells, and the extent of the transformation, depended on the specific ErbB4 signaling pathway that was targeted (Fig. 6*B*–*H*). We first examined the morphology of normal (type 1 and type 2; Fig. 6*I*) MGE cells and found a significant increase in the proportion of branched/bifurcated (type 2) cells when they overexpressed intact ErbB4 (Cyt1; type 1: 25 ± 1%; type 2: 75 ± 1%, 44 cells; Fig. 6*I*) or genetically modified ErbB4 (Cyt1^T1152A^; type 1: 28 ± 2%; type 2: 72 ± 2%, 37 cells; Cyt1^Y1056F^; type 1: 25 ± 3%; type 2: 75 ± 1%, 30 cells, Cyt2: type 1: 27 ± 2%; type 2: 73 ± 3%, 49 cells, Cyt2T1152A; type 1: 25 ± 1%; type 2: 75 ± 2%, 45 cells; Fig. 6*I*) compared with control (pCAG; type 1: 39 ± 2%; type 2: 61 ± 1%, 39 cells; Fig. 6*I*). On the other hand, cells that expressed ErbB4ΔICD and found at the PSB exhibited morphology similar to pCAG control (ErbB4ΔICD; type 1: 42 ± 1%; type 2: 58 ± 1%, 21 cells; Fig. 6*I*). Interestingly, brush-like branched (type 3) and round (type 4) cells were never observed after transfection with control plasmid (pCAG) or ErbB4ΔICD. However, the proportion of brush-like branched (type 3) cells per entire polar cell population (type 1–3; Fig. 6*J*) or the proportion of round (type 4) cells per total number of examined cells (type 1–4; Fig. 6*K*) was significantly increased when MGE cells overexpressed intact or genetically modified ErbB4. The extent of phenotypic change depended on the ErbB4 signaling pathway(s) affected: mild (Cyt1; type 3: 12 ± 2%; type 4: 7 ± 1%, 232 cells), moderate (Cyt1^T1152A^; type 3: 23 ± 2%; type 4: 13 ± 2%, 298 cells) or severe (Cyt1^Y1056F^; type 3: 33 ± 3%; type 4: 22 ± 1%, 212 cells; Cyt2; type 3: 32 ± 4%; type 4: 26 ± 2%, 145 cells; Cyt2^T1152A^; type 3: 33 ± 2%; type 4: 25 ± 2%, 283 cells) (Fig. 6*J*–*L*). In summary, abolition of ErbB4 phosphorylation by Cdk5 on residue T1152 had a profound effect on polarity and directionality of MGE cells. Furthermore, deficiency of both phosphorylation-sensitive residues (Cdk5-targeted T1152 and PI3-kinase-binding Y1056 in Cyt2^T1152A^) had the same great impact as loss of Y1056 alone (Cyt1^Y1056F^ and Cyt2) (Fig. 5*L*), confirming that Cdk5 regulates ErbB4/PI3-kinase activity in migrating interneurons (see Fig. 4*D*).

### Loss of *p35* and *ErbB4* Alter the Number of Interneurons That Reach the Cx via Different Mechanisms

**Figure 7.**
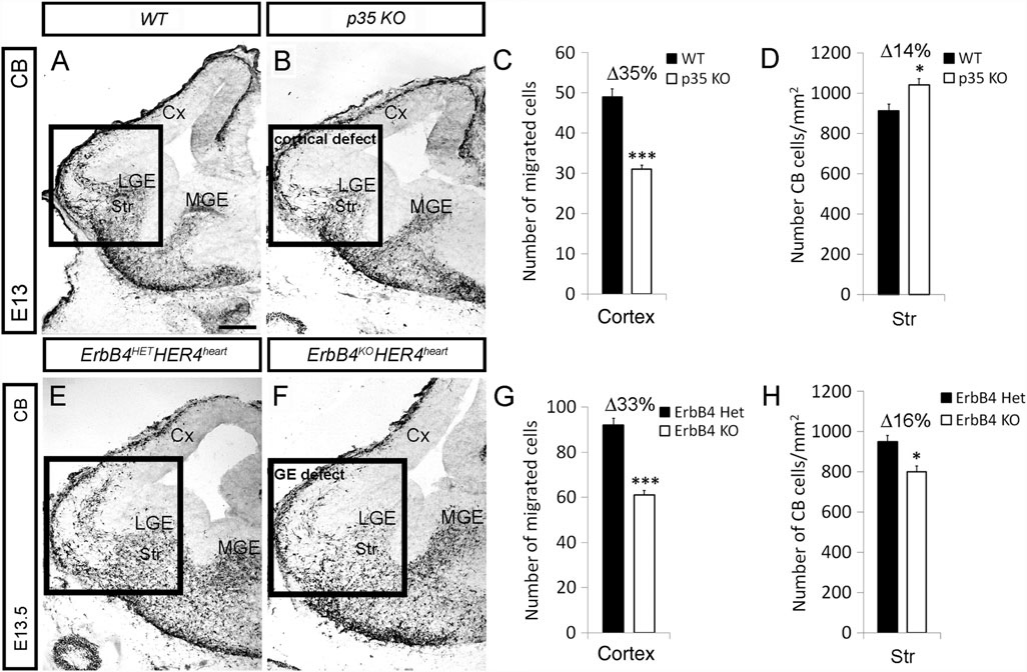
Loss of *p35* and *ErbB4* alter the number of interneurons that reach the Cx via different mechanisms. (*A*, *B*, *E*, *F*) Embryonic forebrain sections of indicated genotypes and ages, immunostained for CB. The LGE/Cx junction is shown within a box outline. (*C*, *D*, *G*, *F*) Quantification of the number of CB cells in the Cx and striatum (Str) of control (black) and indicated KO (white) animals. *P ≤ 0.05, ***P ≤ 0.005, *t*-test. Bar, 200 μm.

**Figure 8.**
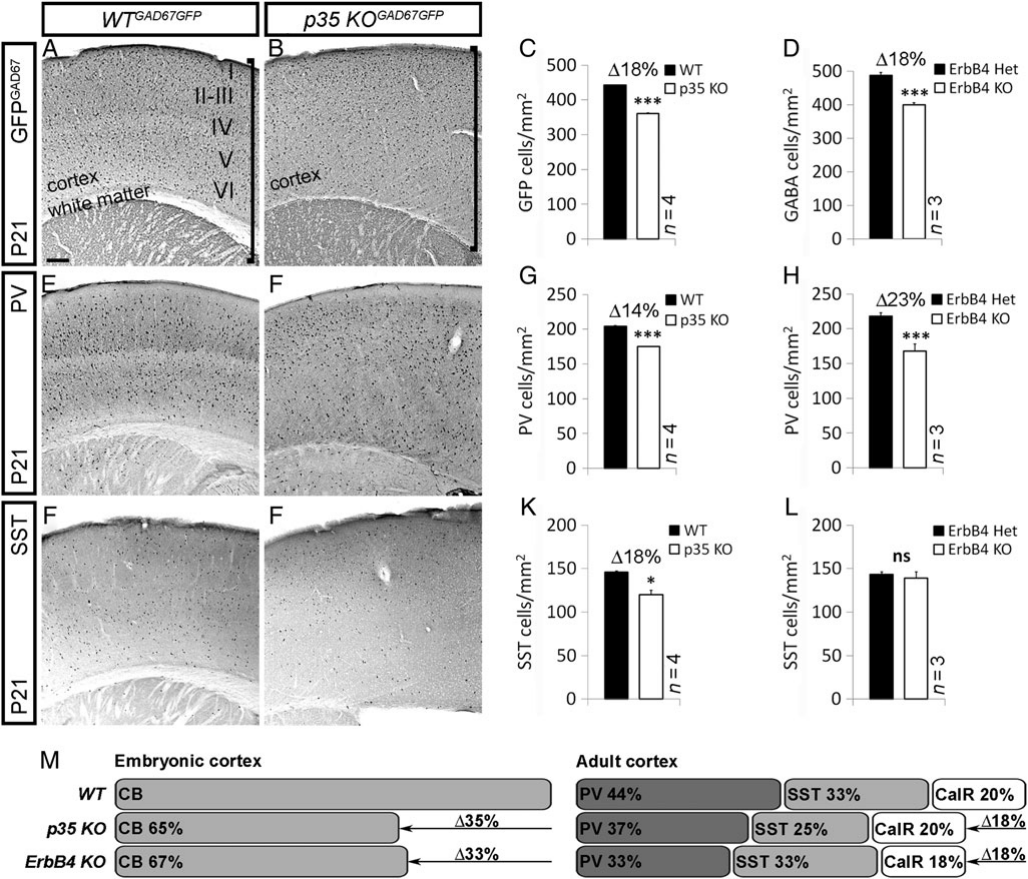
Loss of *p35* and *ErbB4* results in reduction of a subset of interneurons in the adult Cx. (*A*, *B*, *E*, *F*, *I*, *J*) Sections of adult (P21) somatosensory Cx of control and *p35* KO mouse, immunostained for GFP, PV, and SST, indicating the quantity and distribution of interneurons. (*A*, *B*) Vertical line specifies the width of the Cx and white matter. Roman numerals designate cortical layers in control animals. Loss of *p35* alters cortical laminar organization (layers are inverted compared with controls), and white matter is histologically undetectable due to misguided fibers that run through the center of the Cx (Rakić et al. 2006). (*C*, *D*, *G*, *H*, *K*, *L*) Quantification of the number of GFP^GAD67^, PV, and SST interneurons in the Cx of control (black) and indicated KO (white) animals. *P ≤ 0.05, ***P ≤ 0.005, *t*-test. (*M*) Schematic, showing loss of embryonic and postnatal interneurons in *p35* and *ErbB4* KOs compared with controls. Bar, 50 μm.

Next, we compared interneuron phenotype in the developing Cx of *p35* and conditional *ErbB4HER4^heart^* KOs by using immunohistochemistry with calbindin (CB) ab. In both KO mice we detected significantly fewer CB^+^ cells in the Cx compared with control animals (*WT*: 49 ± 2 vs. *p35* KO: 31 ± 1 at E13; *ErbB4HER4^heart^*, *HET*: 92 ± 3 vs. KO: 61 ± 2 at E13.5; Figs 7*A*–*C*, *E*–*G* and 8*M*). However, the number of CB^+^ cells in the striatum at E14.5 was increased in *p35*- and reduced in *ErbB4*-lacking animals compared with control littermates (*WT*: 913 ± 33 vs. *p35 KO*: 1043 ± 30; *ErbB4HER4^heart^*, *HET*: 951 ± 31 vs. KO: 801 ± 29; Fig. 7*D*,*H*). Therefore, in *p35* KOs, interneurons failed to cross the PSB and exhibited a “cortical migratory defect,” while in *ErbB4* conditional KOs these cells could not advance through the LGE/striatum, thus showing a “GE migratory defect” (see Fig. 5*K*). Besides, the levels of ErbBs, ligands and associated molecules, assessed in a microarray study, remain normal in embryonic *p35* KOs (Supplementary Table 2), as well as cell proliferation and cell death in the embryonic forebrain in both the *p35* and *ErbB4HER4^heart^* KOs (Supplementary Fig. 5).

### Loss of *p35* and *ErbB4* Results in Reduction of a Subset of Interneurons in the Adult Cx

Lastly, to determine whether reduction of interneurons in the developing Cx of *p35* and *ErbB4HER4^heart^* KOs was permanent, we looked into numbers of adult cortical GABAergic neurons. Immunohistochemical analysis of mice at P21, using GFP (in *p35^GAD67GFP^* litters) or GABA (in *ErbB4HER4^heart^* litters) ab, revealed a significant decrease in the density of GABAergic cells in both the *p35* and the *ErbB4* KOs compared with controls (*p35*: 361 ± 1 vs. 442 ± 1; Fig. 8*A*–*C*; *ErbB4HER4^heart^*: 399 ± 7 vs. 487 ± 9; Fig. 8*D*). We next wondered what specific classes of interneurons were affected by loss of *p35* or *ErbB4*, and discovered that these 2 genes could control the development of the same, but also different interneuron subpopulations. While there was a significant reduction in density of PV-containing interneurons in both KOs compared with control littermates (*p35*: 175 ± 1 vs. 204 ± 1; Fig. 8*E*–*G*,*M*; *ErbB4HER4^heart^*: 168 ± 10 vs. 218 ± 15; Fig. 8*H*,*M*), we observed fewer SST^+^ cells only in *p35* KO animals compared with controls (120 ± 5 vs. 146 ± 1; Fig. 8*I*–*L*,*M*). In addition, loss of either *p35* or *ErbB4* gene did not affect the calretinin (CalR) interneuron numbers (data not shown; Fig. 8*M*) or specification of interneuron subtypes (Supplementary Fig. 6).

## Discussion

We discovered that cortical interneurons utilize specific ErbB4-mediated signaling pathways to regulate their motility while coursing toward and through forebrain areas enriched in NRG1, NRG3, and HB-EGF. Explicitly, we found that p35/Cdk5 plays an important role in their directed migration by phosphorylating ErbB4 and subsequently modulating one of the canonical ErbB4 signaling pathways, PI3-kinase/Akt.

### Differential Expression of ErbB4 Isoforms in Migrating Cortical Interneurons

We found that ErbB4 is the only ErbB family member expressed by migrating cortical interneurons. This gene encodes 2 distinct cytoplasmic isoforms that can selectively regulate growth factor responses by activating different signaling pathways (Junttila et al. 2000). Published evidence suggests that isoform Cyt1, unlike Cyt2, activates the PI3-kinase/Akt pathway; both isoforms can stimulate the MAPK pathway (Sundvall et al. 2008).

Interneurons born in the MGE migrate through the subpallium to reach the Cx and differentiate predominantly into PV- and SST-positive subtypes (Fishell and Rudy 2011). We showed that the Cyt2 isoform is present in interneurons as they leave the MGE and travel through a permissive corridor containing LGE-derived cells that express a membrane-bound NRG1-CRD (type III) (Calaora et al. 2001; Assimacopoulos et al. 2003; Flames et al. 2004; López-Bendito et al. 2006). Therefore, Cyt2 is most likely required for their migration through the subpallium by haptotaxis via cell-to-cell interactions with NRG1-CDR^+^ cells (Supplementary Fig. 8 and Table 3). On the other hand, early-born migrating interneurons (E12.5/13.5) do not express Cyt1 until they reach the PSB, a cortical signaling center also known as antihem (Assimacopoulos et al. 2003), the “angle” region (López-Bendito et al. 2006) or the ventral pallium (Subramanian et al. 2009), where they encountertwo ErbB4 ligands, NRG1-Ig (secreted; type I and II; Flames et al. 2004) and NRG3 (Assimacopoulos et al. 2003). Therefore, Cyt1 regulates interneuron-directed chemotaxis toward cortical sources of ErbB4 ligands (Supplementary Fig. 8 and Table 3). Interneurons with altered ErbB4/PI3-kinase activity, as shown in this study, can pass through the MGE/LGE permissive corridor, but their migration is random, the leading process anomalous, and many cannot traverse the PSB. Yet, in the event of complete loss of ErbB4 or NRG1 signaling in the developing forebrain, a proportion of these cells fail to advance and stay confined to the MGE (this study; Flames et al. 2004). Therefore, distinct ErbB4 pathways are required for tangential interneuron migration through the developing forebrain (Supplementary Fig. 8 and Table 3). To further address the exact role of different ErbB4 isoforms and accompanied signaling pathways in interneuron migration, electroporation of *ErbB4HER4^heart^* animals with either Cyt1 or Cyt2 should be performed.

### ErbB4 as a Cdk5 Substrate

We provide the first evidence that Cdk5 phosphorylates ErbB4 and facilitates cell migration by modifying ErbB4 signaling. Other ErbBs are known substrates of Cdk5; specifically, Cdk5 phosphorylates ErbB2 and ErbB3 at the neuromuscular junction (NMJ; Fu et al. 2001, 2005) and in perinatal cortical neurons (Li et al. 2003) regulating neurotransmission and neuronal survival, respectively.

Cdk5 phosphorylates ErbB4 at T1152, situated in close proximity to the PI3-kinase-binding site (Y1056), and in turn promotes ErbB4 tyrosine phosphorylation. Loss of Cdk5 function suppresses ErbB4 Y1056 phosphorylation and subsequently PI3-kinase activity. Recent studies have also shown that p35/Cdk5 positively controls ErbB2 (Fu et al. 2005) and PI3-kinase/Akt (Li et al. 2003) kinase activity by phosphorylating ErbB2/ErbB3. In contrast, phosphorylation of AATYK1 (apoptosis-associated tyrosine kinase 1) by Cdk5 represses its tyrosine phosphorylation (Tsutsumi et al. 2010). It is not clear how exactly Cdk5 controls tyrosine phosphorylation, directly, by changing the conformation of ErbB4, or via other kinases (e.g., Src family kinases; Tsutsumi et al. 2010). Nevertheless, our study demonstrates the importance of ErbB4 phosphorylation by Cdk5 in cortical interneuron migration (Supplementary Fig. 8).

### ErbB4, PI3-Kinase, Cdk5, and Cytoskeleton in Neuronal Migration

In cortical interneurons, leading process dynamics and, accordingly, cell polarity and directionality most likely rely on a microtubule network (Baudoin et al. 2008), while nuclear translocation toward the selected leading process and, consequently, locomotion are primarily driven by actomyosin forces applied to the rear of the nucleus (Bellion et al. 2005; Martini and Valdeolmillos 2010). We have previously demonstrated that lack of Cdk5 activity in *p35* KOs alters leading process morphology in migrating cortical interneurons. Specifically, *p35*-deficient interneurons display a significant increase in branching while entering the Cx from ventral telencephalic sources. Similarly, motor axon projections at the NMJ (Fu et al. 2005) and migrating pyramidal neurons in the Cx (Gupta et al. 2003; Ohshima et al. 2007) have profuse and anomalous branching patterns in *p35*- or *Cdk5*-KOs. Furthermore, *p35*-deficient interneurons or pyramidal cells exhibit impaired migration (this study; Gupta et al. 2003; Rakić et al. 2009).

Here, we present original evidence that interdependent signaling pathways, PI3-kinase and Cdk5, play a key role in ErbB4-mediated interneuron migration. Namely, alteration in ErbB4 signaling, due to lack of PI3-kinase binding, Cdk5 phosphorylation or both, impair directionality and leading process morphology, and almost completely mimics the phenotype seen when interneurons are treated with nocodazole, a microtubule destabilizing drug (Baudoin et al. 2008). The majority of affected cells either 1) have a leading process that is shortened and thickened with numerous tiny protrusions (brush-like) or 2) lose their polarity and exhibit a round and multipolar appearance. In contrast, a small percentage of cells maintained normal polarity and neurite length, but were seen to bifurcate more extensively; this is reminiscent of the phenotype observed when *Doublecortin*, a microtubule stabilizing protein, is knocked out (Kappeler et al. 2006; Friocourt et al. 2007). Therefore, different alterations in pathways controlling microtubule stability may lead to diverse abnormalities in interneuron leading process dynamics.

Loss of *ErbB4* or *p35* in mice gives rise to a similar phenotype characterized by alteration in tangential migration and a reduction in the number of interneurons in the adult Cx (this study; Flames et al. 2004; Rakić et al. 2009). This phenotype is, however, achieved by distinct mechanisms; ErbB4 (Cyt2 isoform) is needed for MGE to LGE interneuron migration via yet unknown signaling mechanism(s), while p35/Cdk5 plays a role in ErbB4-mediated migration as interneurons enter the Cx and express the Cyt1 isoform. It is possible that Cdk5-dependent PI3-kinase activity in ErbB4-expressing interneurons is necessary to determine direction of their movement, but also to mediate nucleokinesis, as some cells (∼20%) never make it to the Cx in *p35* KOs. In line with this, it has been shown that PI3-kinase regulates small Rho GTPases (Waite and Eickholt 2010) and, yet, RhoA/ROCK signaling contributes to cell polarity and migration by affecting both microtubule stability and actomyosin contractility (Takesono et al. 2010). Interestingly, ErbB4 has been found in close proximity to microtubules in adult interneurons (Mechawar et al. 2007). Here, we observed that ErbB4 co-localizes with β-III-tubulin, a neuron-specific tubulin that makes up microtubules, in migrating interneurons. Further electron microscopic and biochemical binding studies are needed to reveal whether ErbB4 directly associates with the cytoskeleton and/or cytoskeletal regulatory molecules, such as p35/Cdk5, during the process of cytokinesis.

In summary, our results illustrate the importance of co-activation of PI3-kinase/Akt and p35/Cdk5 signaling pathways at the interface between pallium and subpallium in supporting migration of ErbB4^+^ interneurons towards cortical sources of ErbB4 ligands. The abnormal interneuron migration seen in KO mice lacking either *ErbB4* or *p35* results in a permanent absence of certain interneuron subtypes (PV^+^ or PV^+^ and SST^+^ cells, respectively) which may promote the formation of aberrant neuronal circuitry underpinning clinically-defined neurodevelopmental disorders. Indeed, interference in NRG1/ErbB4 and p35/Cdk5 signaling has been associated with epilepsy (Dhavan and Tsai 2001; Li et al. 2011; Tan et al. 2011) and schizophrenia (Engmann et al. 2011; Marín 2012), in both mice and humans. Here, we have provided strong evidence of a link between the 2 pathways with respect to interneuron migration. Both ErbB4 and Cdk5 signaling are critical players in synapse formation, and further investigation of their interaction in this respect is likely to be important for the elucidation of pathological circuitries associated with different clinical correlates.

## Supplementary Material

Supplementary material can be found at: http://www.cercor.oxfordjournals.org/.

## Funding

This work was supported by Wellcome Trust (grant numbers 074549 and 089775; J.G.P.), the Strategic Research Program for Brain Sciences (“Understanding of molecular and environmental bases for brain health”; K.N.), the Grant-in-Aid for Scientific Research of the Ministry of Education, Culture, Sports, Science, and Technology of Japan (K.N.) and grants from Breakthrough Breast Cancer (B.H.). Funding to pay the Open Access publication charges for this article was provided by the Wellcome Trust.

## Supplementary Material

Supplementary Data
