# Supplementary material for: Cdk5 Phosphorylation of ErbB4 is Required for Tangential Migration of Cortical Interneurons
Source: Cereb Cortex. 2013 Oct 18;25(4):991–1003. doi: 10.1093/cercor/bht290 (PMC4380000; doi:10.1093/cercor/bht290)
Supplement: Supplementary Data [file supp_25_4_991__index.html]

Cdk5 Phosphorylation of ErbB4 is Required for Tangential Migration of Cortical Interneurons — Cdk5 Phosphorylation of ErbB4 is Required for Tangential Migration of Cortical Interneurons — Supplementary Data 

# Cdk5 Phosphorylation of ErbB4 is Required for Tangential Migration of Cortical Interneurons

## Supplementary Data

Supplementary Data

**Files in this Data Supplement:**

- Supplementary Data - Doc file
- Supplementary Figure 1 - jpg file
- Supplementary Figure 2 - jpg file
- Supplementary Figure 3 - jpg file
- Supplementary Figure 4 - jpg file
- Supplementary Figure 5 - jpg file
- Supplementary Figure 6 - jpg file
- Supplementary Figure 7 - jpg file
- Supplementary Figure 8 - jpg file
