# Supplementary material for: Cdk5 Phosphorylation of ErbB4 is Required for Tangential Migration of Cortical Interneurons
Source: Cereb Cortex. 2013 Oct 18;25(4):991–1003. doi: 10.1093/cercor/bht290 (PMC4380000; doi:10.1093/cercor/bht290)
Supplement: Supplementary Data [file supp_bht290_bht290supp.doc]

**SUPPLEMENTARY MATERIAL**

**MATERIALS AND METHODS**

**Mouse lines**

*p35* (Chae et al. 1997), *Cdk5* (The Jackson Laboratory; Ohshima et al. 1996), *glutamate decarboxylase 67 (GAD67)-green fluorescent protein (GFP)* (Δneo) (Tamamaki et al. 2003), ErbB4HER4^heart^ (Tidcombe et al. 2003) and ErbB4^HET^ (Gassmann et al. 1995) mice, maintained in C57/Black6 background, were used in this study. *GAD67-GFP* (Δneo) transgenic mice were crossed with *p35^HET^* to produce *p35^HET^GFP^GAD67^* (Rakic et al. 2009). Likewise, transgenic mice expressing HER4 under the control of the cardiac-specific myosin promoter (ErbB4HER4^heart^) were crossed with ErbB4^HET^ mice to generate ErbB4^HET^HER4^heart^mice (Tidcombe et al. 2003).  Crossing between heterozygous animals produced knockout mice for *p35* and *ErbB4* genes. The day the vaginal plug was found was considered as embryonic (E) day 0.5. All procedures were performed under licence, and in accordance to the regulations of the UK Home Office, UCL Animal Ethics Committee, Japan Neuroscience Society and Keio University School of Medicine.

**Cell lines and transfection**

**GN11 (Cariboni et al., 2005)** and COS7 cells were grown in Dulbecco’s modified Eagle medium supplemented with 10% foetal bovine serum, 500 µM L-glutamine and penicillin/streptomycin, all from Invitrogen, Paisley, UK. Cells were transfected with expression vectors using Lipofectamine 2000 reagent (Invitrogen, Paisley, UK) according to manufacturer’s protocol and collected after 48 hours.

**Fluorescence-activated cell sorting (FACS) of transfected GN11 cells**

**48 hours after transfection, GN11 neurons were trypsinized, pelleted by centrifugation at 1,000 x g for 3 minutes, resuspended in 2 ml of medium, and cell numbers were estimated using a haemocytometer. The cells were spun down again at 1,000 x g for 3 minutes, and 10-12x10^6^ cells were resuspended in 1 ml of medium, and transferred in 5 ml Falcon tubes to the FACS sorting machine (MoFlo XDP, Beckman Coulter, High Wycombe, UK). GFP^+^ cells were collected into tubes, and prepared for chemotaxis experiments.**

**Total RNA extraction, reverse transcription, RT-PCR and primers**

RNA was isolated using RNeasy Plus Mini (whole cortex **and GN11 cells**) or RNeasy Micro (FACS-purified forebrain cells) kit (Qiagen, Chatsworth, CA) according to the manufacturer’s protocol. RNA was treated with DNase I (Amplification grade; Invitrogen) to remove any residual DNA and used for RT-PCR or microarray analysis. cDNA for RT-PCR was generated from either 1 μg of RNA using avian myeloblastosis virus reverse transcriptase and (Promega, Southampton, UK; whole cortex) or 20 ng of RNA using the Whole Transcriptome Amplification Kit (Qiagen; FACS-purified forebrain cells) as described in the manufacturer’s protocols. The list of primers used in this study can be found in the Table S1.

**Microarray analysis**

Total RNA (100 ng) from mouse E15.5 cortex, GFP-positive FACS-purified forebrain cells (E13.5 and E15.5; *GAD67-GFP* transgenic mice) or **GN11 cells** was assessed for the quality, converted to single-strand (sense) cDNA and subsequently used for microarray hybridisation by utilising the Mouse Gene 1.0_ST Gene-Chip array (Affymetrix, High Wycombe, UK) as described previously (Faux et al., 2010). Analysis of DNA microarray data was performed using either Affymetrix Expression Console or GeneSpring GX (Agilent Technologies, Wokingham, UK) software.

**Antibodies, drugs and recombinant peptides**

The following antibodies, in alphabetical order, were used in immunoblotting (IB) and immunohistochemistry (IHC): Akt (rabbit, 1/1000, Cell Signaling Technology, Danvers, MA), phospho-Akt-Ser373 (rabbit, 1/1000, Cell Signaling Technology), β-actin (mouse, 1/5000, Sigma-Aldrich, St. Louis, MO), active caspase 3 (rabbit, 1/250, R&D Systems, Minneapolis, MN), calbindin (CB; rabbit, 1/5000, SWant, Switzerland), calretinin (CalR; rabbit, 1/2000, SWant), Cdk5 (mouse, clone DC27, 1/5, a kind gift of LH Tsai), ErbB3 (rabbit, 1/1000, Santa Cruz Biotechnology, Santa Cruz, CA), ErbB4 (C283; rabbit, IB: 1/1000, Santa Cruz Biotechnology), ErbB4 (0618; rabbit, IHC: 1/100, a kind gift of C Lai), phospho-ErbB4-Tyr1056 (rabbit, IB: 1/1000, IHC: 1/100, Santa Cruz Biotechnology), phospho-ErbB4-Thr1152 (rabbit, IB: 1000, IHC: 1/100, custom-made by Sigma-Genosys, Haverhill, UK), GFP (rabbit, 1/5000, Abcam, Cambridge, UK), phospho-histone 3 (pH3; rabbit, 1/1000, Millipore, Watford, UK), myc (mouse, clone 9E10, 1/5000, Sigma-Aldrich), neuregulin (NRG) 3 (EGF-like domain: Glu284-Pro344, goat, IHC: 1/50, dissociated neurons: 100 μg/ml for 30 minutes prior to the chemotactic assay, R&D Systems), NRG3 (cleaved region: His347-Val363, rabbit, 1/50, Abgent, San Diego, USA), p35 (C19; rabbit, 1/1000, Santa Cruz Biotechnology), parvalbumin (PV; mouse, 1/2000, SWant), somatostatin (SST; rabbit, 1/200, Millipore), phospho-Thr-Pro (pTP; mouse clone 42H2, 1/4000, Cell Signaling Technology), β-III-tubulin (mouse, clone SDL.3D10, 1/500, Sigma-Aldrich).

Roscovitine, a Cdk5 kinase inhibitor, and LY294002, a PI3-kinase inhibitor, were purchased from Sigma-Aldrich, dissolved in DMSO (concentration: 10 mM) and kept at -20^o^C. Cells were treated with 10 μM kinase inhibitor for 30 minutes prior to the chemotactic assay.

Recombinant human NRG-1β1_176–246_ (EGF-like domain) and HB-EGF_1-148_ (including the EGF-like domain_108-148_), both obtained from R&D Systems, dissolved in sterile PBS supplemented with bovine serum albumin, and kept at -80^0^C.

**Immunohistochemistry**

Brain tissue was fixed in 4% PFA/PBS for 2 hours, transferred to 30% sucrose, cryosectioned at 20-40 µm, and immunolabelled, as described previously (Rakic et al. 2006; Rakic et al. 2009), with antibodies in PBS containing blocking solution (DAKO, Ely, UK) overnight at room temperature. The proteins were then visualised by standard immunoperoxidase and immunofluorescence methods.

**Cloning**

JMa-Cyt1 (referred to as Cyt1) and JMa-Cyt2 (referred to as Cyt2) isoforms of ErbB4, as well as ErbB4ΔICD-JMa (referred to as ErbB4ΔICD), truncated for most of the intracellular domain (ICD), were cloned from a rat adult forebrain cDNA library, using the proofreading DNA polymerase *Pfu.* ErbB4ΔICD-JMa, truncated for most of the intracellular domain (ICD) including the tyrosine kinase domain, was cloned first with the following primers cagt*gggccc*cacgaattctgagacttgc and cagt*tctaga*cccgagccaaggacttttac, and resulting PCR product was ligated into pcDNA3.1mychis B (-) (Invitrogen) between the *Apa* I and *Xba* I sites. The Cyt1 and Cyt2 intracellular isoforms of ErbB4 were then cloned with the following primers GGACCTGACAAC*TGTACA*AAGTGT and CAGT*TCTAGA*GC**T**CTCACCACAGTATTCC, and resulting PCR products were ligated into the *BsrG* I and *Xba* I sites of ErbB4ΔIC-JMa-myc (note that the reverse primer contained the stop codon that was mutated (TGA🡪AGA) in order to permit the expression of the contiguous myc sequence). All myc-tagged constructs were sequenced to confirm their correct identity, and subsequently subcloned into pCAG-IRES2-EGFP vector (Kawauchi et al., 2003) using *Afl* II (*Bst98* I) and *Cla* I blunt-ended sites, respectively, and the *Nhe I* site. The rat ErbB4 isoforms, JMa-Cyt1 (accession number AY375306.1) and JMa-Cyt2 (accession number AY375307.1), submitted to the GenBank by Gambarotta et al. in 2003, entirely matched the sequences obtained in this study.

ErbB4 fragment, encoding T1152-containing peptide (ErbB4_Ala1143-Tyr1262_), was obtained by PCR amplification using the forward primer (GTCAGTCGACAGCAGAACTGGATGAAGA) and the reverse primer (GTCAGCGGCCGCGTATTCCTGCAGGTAGT) and JMa-Cyt1-myc as a template, and ligated into pGEX-4T2 (GE Healthcare, Slough, UK) between *Sal* I and *Not* I sites, to allow bacterial production of gluthatione-S-transferase (GST)-tagged peptide referred to as GST-T. All reagents were purchased from Promega unless otherwise specified.

**Site-directed mutagenesis**

A single point mutation in the Cdk5 phophorylation [T1152 (ACT) to A (GCT)] or PI3-kinase binding site [Y1056 (TAC) to F (TTC)] of ErbB4 or multiple point mutations within the EGF-like domain of Nrg3 [referred as to Nrg3mut: C1 (TGT) to G (GGT), C2 (TGT) to F (TTT), C6 (TGT) to G (GGT) and conserved R (CGT) before C6 to P (CCT)] were introduced using a standard QuikChange^R^ II XL Site-Directed Mutagenesis Kit (Agilent Technologies, Wokingham, UK). Briefly, the entire (JMa-Cyt1-myc or JMa-Cyt2-myc) or fragment (GST-T) ErbB4 was amplified by PCR using oligonucleotides containing one base substitution (ACT 🡪 GCT for T1152A; GAAGAAGGCTACATG**G**CTCCCATGCATGACAAG; TAC 🡪 TTC for Y1056F; CTCCTCCTGCC**T**TCACCCCCATGTCGGGAAATCAG), the template methylated strand was destroyed enzymatically with *Dpn1*, and purified constructs were transformed into bacteria and verified by direct sequencing. After site-directed mutagenesis of the receptor, a short sequence of either JMa-Cyt1-myc containing the mutated PI3K-binding site (Y1056) or JMa-Cyt1-myc and JMa-Cyt2-myc containing the mutated Cdk5 phosphorylation site (T1152), was re-ligated into appropriate JMa-Cyt1-myc or JMa-Cyt2-myc plasmids, using the *Cla* I and *Xho* I or *Xho I* and *Bst98 I* restriction sites, respectively. In JMa-Cyt1-myc clones, the *Cla* I restriction site was protected from cleavage as it was methylated by *Dam* methylase. To prevent *Dam* methylase activity, the plasmids were propagated in a *Dam*-negative bacteria strain before *Cla* I digestion. All reagents were purchased from Promega unless otherwise specified.

Preparation of GST-fusion proteins

Glycerol stocks of BL21 strain of *E. coli* colonies expressing the GST-T1152 and GST-T1152A proteins were picked and inoculated into fresh LB/ampicillin broth, followed by overnight growth at 37^o^C. The following morning, the overnight cultures were diluted into a 10-fold excess of LB/ampicillin broth and incubated at 37^o^C with shaking until the protein absorbance reading (A_560_) reached 0.9-1. At this stage, 1M IPTG (isopropyl β-D-1-thiogalactopyranoside) was added to give a final concentration of 0.5 M in order to induce expression of GST-fusion proteins, and incubation was continued for a further 3 hours with shaking at 37^o^C. The cultures were centrifuged at 10,000 rpm for 10 minutes to pellet the bacteria. The pellet was then resuspended in ErbB4 lysis buffer, containing protease and phosphatase inhibitors, and freeze-thawed in liquid nitrogen 3 times. The suspension was then sonicated on ice 2 x 2 minutes. This was followed by centrifugation at 10,000 rpm for 20 minutes. The protein supernatant was then incubated with gluthation-sepharose beads (10% stock in ErbB4 lysis buffer) for an hour at 4^o^C, after which the beads were washed three times with ErbB4 lysis buffer. In order to elute the protein, the beads were then incubated with 15 mM glutathione in ErbB4 lysis buffer, for 10 minutes at room temperature. To isolate the GST-fusion proteins, elute was dialysed against 3 litre of pre-chilled PBS overnight at 4^o^C. In order to check for protein degradation and for comparison of protein fractions of uninduced and induced cells, 10 ml of the protein supernatant was loaded on a 10% polyacrilamide gel and stained with Coomassie Blue. The protein concentration was determined using the BioRad (Hemel Hempstead, UK) protein assay. The GST-fusion proteins were stored at -80^o^C or used immediately for *in vitro* kinase assay. All reagents were purchased from Sigma-Aldrich unless otherwise specified.

Protein isolation, immunoprecipitation, immunoblotting and kinase assay

Protein isolation, immunoprecipitation, immunoblotting and kinase assay were performed as previously described (Causeret et al. 2007; Jacobs et al. 2007; Rakic et al. 2009). Mouse cortices or transfected COS7 cells were lysed on ice in pre-chilled lysis buffer containing protease (PMSF, aprotinin, leupeptin, pepstatin: Complete-Mini; Roche, Welwyn, UK) and phosphatase inhibitors (sodium fluoride and sodium orthovanadate) for 20 minutes. Two different lysis buffers were used; one for detecting the ErbB receptors (ErbB4 lysis buffer: 50 mM Tris-HCl, pH 8, 150 mM NaCl, 1% Nonidet P-40 and protease/phosphatase inhibitors) and the other for Cdk5 immunoprecipitation (Cdk5 lysis buffer: 25 mM Tris-HCl, pH 7.4, 150 mM NaCl, 5 mM EDTA, pH 8.0, 1% Triton X-100 and 10% glycerol and protease/phosphatase inhibitors). The cell suspension, obtained from cortical tissue or COS7 cells, was centrifuged at 13,000 rpm for 8 minutes at 4^o^C and the supernatant was collected for immunoprecipitation and/or immunoblotting. Protein concentration was determined using the Bio-Rad Protein Assay.

In order to immunoprecipitate p35/Cdk5 complex or myc-tagged ErbB4 from transfected COS7 lysates, and ErbB4 from cortical lysates, 3-5 μl of rabbit anti-p35 or mouse anti-myc antibody, and rabbit anti-ErbB4 antibody, respectively, were incubated with 200-500 μl of lysates at 4^o^C for an hour. 100 μl of Protein A (for rabbit antibodies) or Protein G sepharose (for mouse antibody) beads, prepared as 10% solution in Cdk5 kinase (30 mM HEPES, pH 7.2, 10 mM MgCl_2_, 5 mM MnCl_2_, 1mM DTT) or ErbB4 lysis buffer, were then added, and the suspension was incubated for another hour at 4^o^C on a rocking platform. Immunoprecipitates were washed three times in Cdk5 kinase or ErbB4 lysis buffer, briefly dried with vacuum, resuspended in 50 μl or 10 μl of 1x sample buffer, and used for either immunoblotting or *in vitro* kinase assay.

For immunoblotting, cell lysates were separated by sodium dodecyl sulfate–polyacrylamide gel electrophoresis (SDS–PAGE), transferred onto polyvinylidene difluoride membrane (Immobilon; Millipore, Watford, UK), and blocked in 5% skimmed milk, and incubated with primary antibodies for an hour at room temperature or overnight at 4^o^C. The horseradish peroxidase conjugated secondary antibodies (Vector laboratories, Peterborough, UK) followed by the Enhanced Chemiluminescence reagent (GE Healthcare) was used to visualise immunodetected proteins on a film (Kodak X-OMAT). For kinase assays 2 mg of GST-T1152 and GST-T1152A proteins were incubated with p35/Cdk5-containing sepharose beads supplemented with 50-μM ATP, 1 μCi [32Pγ-ATP] (GE Healthcare), 1 mM dithiothreitol, and Cdk5 kinase buffer (50 mM 4-(2-hydroxyethyl)-1-piperazineethanesulfonic acid, pH 7.5, 10 mM MgCl2) in a final volume of 50 μl for 30 minutes at room temperature. Histone 1 (H1; 2mg, Roche) and GST were used as positive and negative substrate controls, respectively. Non-transfected COS7 cell lysates served as a negative control for the Cdk5 kinase assay. The reactions were terminated with 2x sample buffer, resolved by SDS-page and (^32^P)γATP incorporation was assessed by autoradiography. All reagents were purchased from Sigma-Aldrich unless otherwise specified.

***In vitro* chemotactic assay**

Chemotaxis, a migratory response of cells to chemotropic gradient, was studied using a modified 48-well Boyden chamber (Neuro Probe, Gaithersburg, MD) as described previously (Cariboni et al. 2005; Rakic et al. 2009). The upper chamber, containing either E13.5 MGE-derived, E13.5 cortical-derived or **transfected/FACS-purified GFP-positive GN11** cells (10^5^ cells per well), and the lower chamber, filled with recombinant human NRG1β (EGF-like domain), recombinant human HB-EGF (EGF-like domain) or conditioned medium of COS7 transfected with plasmid encoding either mouse NRG3 (EGF-like domain), untreated or treated with a blocking NRG3 antibody (R&D Systems; 100 μg/ml, 30 minutes) that specifically recognises the EGF-like domain of the ligand, or mutated NRG3 (NRG3mut; carries four mutations within the EGF-like domain), were separated by a PVP-free polycarbonate filter (pore size 8 μm). The porous filters were coated with poly-L-lysine:laminin/H_2_O (MGE and cortical cells; 10mg/ml:10mg/ml). Cortical and **GN11** cells were then incubated at 37^o^C in a 5% CO_2_ humidified incubator for 6 and 3 h and fixed in pre-warmed 4% PFA and pre-chilled methanol (Reastain Quick-Diff Fix, Reagena, Finland), respectively. Subsequently, the cells that had migrated were stained using Tiazin and Eosin (Reastain Quick-Diff Red and Blue, Reagena). The images of three fields from each well were collected using a digital camera, and the number of migrated cells was counted in each filed. Results were expressed as mean number of migrated cells per well ± standard error of the mean (SEM). All reagents were purchased from Sigma-Aldrich unless otherwise specified.

**Focal electropration and hemisphere culture**

Focal electroporation and hemisphere culture were performed as reported previously (Kanatani et al. 2008). Briefly, telencephalic hemispheres from E13.5 ICR mouse embryos were dissected out in PBS. The ErbB4 construct vectors were mixed with a pCAG-tdTomato expression vector so that the ratio of the vectors to the pCAG-tdTomato expression vector would be 1:1. To monitor the injection site, a Fast Green solution (0.1%; Sigma) was added to the plasmid solution in a ratio of 1:10 as reported previously (Tabata and Nakajima 2001;Yozu et al. 2005). Approximately 0.1 μl of the plasmid solution (5 μg/μl) was injected into the MGE with a glass micropipette. The telencephalic hemispheres were placed between a platinum plate electrode and a tungsten needle electrode. Electronic pulses (100 V, 5 ms) were discharged four times, 5 ms apart, with an electroporator (CUY21E; NepaGene), and this series of four electroporations was repeated three times for each hemisphere.

Electroporated E13.5 telencephalic hemispheres were cultured for 48 hours. Brains were rotated in 2 ml of DMEM nutrient mixture/Ham's F-12 (Sigma-Aldrich) containing the N-2 supplement (Invitrogen) under a continuous gas flow (95% O2 and 5% CO2 at 37°C) (Nagata and Terashima 1994). Images of the whole-mount telencephalic hemispheres were acquired with a colour cooled CCD camera (VB-7010; KEYENSE) connected to a fluorescent dissecting microscope (M205 FA; Leica).

**Bright field and confocal microscopy**

Immunopreroxidase reacted samples were viewed with the Leica DMR microscope and images captured with the Leica DC 500 digital camera. Immunofluorescence reacted as well as electroporated samples were excited, viewed and photographed under a Leica TCS SP2 confocal laser microscope. All images were finally processed in Adobe Photoshop CS5.

**TEXT**

**To further investigate the potential interaction between PI3-kinase and Cdk5 pathways in NRG1/ErbB4-dependent chemotaxis, we performed an *in vitro* chemotactic assay using GN11 cells, a model cell line obtained from a mouse olfactory bulb tumour with highly migratory behaviour (Maggi et al., 2000; Cariboni et al., 2005), that naturally do not express ErbB4 or the Cdk5 activator p35 as revealed by RT-PCR (Supplementary Fig. S7) and microarray analysis (Table S2). GN11 cells were transfected with single (control-pCAG, Cyt1 or Cyt2) or multiple (ErbB4 isoform plus p35 and Cdk5) GFP-tagged plasmids. GFP^(+)^ cells were then selected by FACS sorting and their potential to migrate towards NRG1β analysed using an *in vitro* chemotactic assay. Both Cyt1 and Cyt2 stimulated chemotaxis compared to control pCAG-expressing cells (pCAG: 70±2; Cyt1: 150±7; Cyt2: 100±3; Supplementary Fig. S7); however, Cyt1 (Supplementary Fig. S7) was much more potent isoform in inducing chemotaxis than Cyt2 (Supplementary Fig. S7). Importantly, in co-transfection experiments, the presence of p35 and Cdk5 significantly increased the chemotactic response of cells expressing Cyt1 (185±5; Supplementary Fig. S7), but not Cyt2 (113±2; Supplementary Fig. S7) compared to single Cyt1 (150±7) or Cyt2 (100±3) transfection. These findings suggest that p35/Cdk5 control chemotaxis via direct regulation of the NRG1/ErbB4/PI3-kinase pathway.**

**REFERENCES**

Chae T, Kwon YT, Bronson R, Dikkes P, Li E, Tsai LH. 1997. Mice lacking p35, a neuronal specific activator of Cdk5, display cortical lamination defects, seizures, and adult lethality. Neuron. 18: 29-42.

Causeret F, Jacobs T, Terao M, Heath O, Hoshino M, Nikolic M. 2007. Neurabin-I is phosphorylated by Cdk5: implications for neuronal morphogenesis and cortical migration. Mol Biol Cell. 18: 4327-4342.

Cariboni A, Rakic S, Liapi A, Maggi R, Goffinet A, Parnavelas JG. 2005. Reelin provides an inhibitory signal in the migration of gonadotropin-releasing hormone neurons. Development. 132: 4709-4718.

Gambarotta G, Garzotto D, Destro E, Mautino B, Giampietro C, Cutrupi S, Dati C, Cattaneo E, Fasolo A, Perroteau I. 2004. ErbB4 expression in neural progenitor cells (ST14A) is necessary to mediate neuregulin-1beta1-induced migration. J Biol Chem. 279: 48808-48816.

Gassmann M, Casagranda F, Orioli D, Simon H, Lai C, Klein R, Lemke G. 1995. Aberrant neural and cardiac development in mice lacking the ErbB4 neuregulin receptor. Nature 378: 390-394.

Jacobs T,  Causeret F,  Nishimura YV, Terao M, Norman A, Hoshino M, Nikolić M. 2007. Localized activation of p21-activated kinase controls neuronal polarity and morphology. J Neurosci. 27: 8604-8615.

Kanatani S, Yozu M, Tabata H, Nakajima K. 2008. COUP-TFII is preferentially expressed in the caudal ganglionic eminence and is involved in the caudal migratory stream. J Neurosci. 28: 13582-13591.

Kawauchi T, Chihama K, Nabeshima Y, Hoshino M. 2003. The in vivo roles of STEF/Tiam1, Rac1 and JNK in cortical neuronal migration. EMBO J. 22: 4190-4201.

Kornblum HI, Zurcher SD, Werb Z, Derynck R, Seroogy KB. 1999. Multiple trophic actions of heparin-binding epidermal growth factor (HB-EGF) in the central nervous system. Eur J Neurosci. 11: 3236-3246.

**Maggi R, Pimpinelli F, Molteni L, Milani M, Martini L, Piva F. 2000. Immortalized luteinizing hormone-releasing hormone neurons show a different migratory activity in vitro. Endocrinology 141:2105-2112.**

Nagata I, Terashima T. 1994. Migration behavior of granule cells on laminin in cerebellar microexplant cultures from early postnatal reeler KO mice. Int J Dev Neurosci. 12: 387-395.

Ohshima T, Ward JM, Huh CG, Longenecker G, Veeranna, Pant HC, Brady RO, Martin LJ, Kulkarni AB. 1996. Targeted disruption of the cyclin-dependent kinase 5 gene results in abnormal corticogenesis, neuronal pathology and perinatal death. Proc Natl Acad Sci U S A. 93: 11173-11178.

Rakić S, Davis C, Molnár Z, Nikolić M, Parnavelas JG. 2006. Role of p35/Cdk5 in preplate splitting in the developing cerebral cortex. Cereb Cortex. 16: Suppl 1:i35-45.

Rakić S, Yanagawa Y, Obata K, Faux C, Parnavelas JG, Nikolić M. 2009. Cortical interneurons require p35/Cdk5 for their migration and laminar organization. Cereb Cortex. 19: 1857-1869.

Tabata H, Nakajima K. 2001. Efficient in utero gene transfer system to the developing mouse brain using electroporation: visualization of neuronal migration in the developing cortex. Neuroscience. 103: 865-872.

Tamamaki N, Yanagawa Y, Tomioka R, Miyazaki J, Obata K, Kaneko T. 2003. Green fluorescent protein expression and colocalization with calretinin, parvalbumin, and somatostatin in the GAD67-GFP knock-in mouse. J Comp Neurol. 467: 60-79.

Tidcombe H, Jackson-Fisher A, Mathers K, Stern DF, Gassmann M, Golding JP. 2003. Neural and mammary gland defects in ErbB4 knockout mice genetically rescued from embryonic lethality. Proc Natl Acad Sci U S A. 100: 8281-8286.

Yozu M, Tabata H, Nakajima K. 2005. The caudal migratory stream: a novel migratory stream of interneurons derived from the caudal ganglionic eminence in the developing mouse forebrain. J Neurosci. 25: 7268-7277.

Zhang D, Sliwkowski MX, Mark M, Frantz G, Akita R, Sun Y, Hillan K, Crowley C, Brush J, Godowski PJ. 1997. Neuregulin-3 (NRG3): a novel neural tissue-enriched protein that binds and activates ErbB4. Proc Natl Acad Sci U S A. 94: 9562-9567.

**TABLES, FIGURE LEGENDS AND FIGURES**

**Table S1** Primer sequences, PCR product size, and NCBI accession number

| **Gene** | **Primer sequence** | **PCR product size** | **NCBI accession number** |
| --- | --- | --- | --- |
| ***ErbB1*** | forward CTGCAGAGGATGTACAACAACTGT  reverse GTTTGTCCCATAGTTGGACAGGAT | 234 bp | NM_207655.2 |
| ***ErbB2*** | forward TGATCATCATGGAGCTGGCGGCC  reverse TTGTCCAAAGGGTCTCGGTTGTC | 382 bp | NM_001003817.1 |
| ***ErbB3*** | forward GGCTCGGGCTCCGACGCTTGTGCT reverse GCAGGGCCGGCACTCATTCTGAGC | 141 bp | NM_010153.1 |
| ***ErbB4*** | forward CAGTACCGAGCCTTGCGCAAATA reverse AGAGTCATGTTGGAAGGCCATGG | 398 bp | AY375306.1 |
| ***ErbB4*-*JM*** | forward GAAATGTCCAGATGGCCTACAG reverse GACAGCAAATGTCAAAGCCATG | *JMa*: 247 bp *JMb:* 208 bp | AY375306.1 |
| ***ErbB4-Cyt*** | forward GAGACCCTCAAAGATACCT reverse TGAGTGCTACTGTCCTCT | *Cyt1:* 442 bp *Cyt2:* 394 bp | AY375306.1 |
| ***p35*** | forward GCCCTTCCTGGTAGAGAGCTG reverse GTGTGAAATAGTGTGGGTCGGC;, | 113 bp | NM_009871.2 |
| ***Cdk5*** | forward GGCTAAAAACCGGGAAACTC reverse CCATTGCAGCTGTCGAAATA | 228 bp | NM_007668.3 |
| ***Actinβ*** | forward CTAAGGCCAACCGTGAAAAG reverse AAGGAAGGCTGGAAAAGAGC | 463 bp | NM_007393.2 |

**Table S2. Expression of ErbBs, their ligands and associated molecules in the** developing mouse forebrain and GN11 cells: A microarray study.

|  | **Gene** | **E15 Cx** | | **GAD67GFP-positive cells** | | | |  |
| --- | --- | --- | --- | --- | --- | --- | --- | --- |
|  |  | ***wt*** | ***p35 KO*** | **E13 Cx** | **E13 GE** | **E15 Cx** | **E15 GE** | **GN11** |
| **Receptor** | *Egfr1/ErbB1* | 99 | 98 | 4 | 4 | 4 | 5 | 43 |
|  | *ErbB2* | 195 | 239 | 5 | 5 | 5 | 5 | 96 |
|  | *ErbB3* | 89 | 101 | 5 | 4 | 4 | 4 | 0 |
|  | *ErbB4* | 809 | 764 | 10 | 10 | 12 | 10 | 0 |
| **Cdk5** | *Cdk5* | 1414 | 1420 | 9 | 9 | 9 | 9 | 149 |
|  | *p35* | 1174 | 583 | 11 | 11 | 11 | 10 | 0 |
|  | *p39* | 214 | 209 | 8 | 8 | 8 | 8 | 0 |
| **ErbB receptor ligands** | *Nrg1^ErbB3/ErbB4^* | 340 | 323 | 5 | 5 | 5 | 5 | 34 |
|  | *Nrg2^ErbB3/ErbB4^* | 119 | 116 | 6 | 6 | 6 | 6 | 0 |
|  | *Nrg3^ErbB4^* | 519 | 523 | 4 | 4 | 4 | 4 | 0 |
|  | *Nrg4^ErbB4^* | 39 | 36 | 4 | 4 | 4 | 4 | 10 |
|  | *HbEgf^ErbB1/ErbB4^* | 274 | 282 | 7 | 6 | 7 | 7 | 215 |
|  | *Tgfa^ErbB1^* | 262 | 244 | 7 | 6 | 7 | 7 | 8 |
|  | *Egf^ErbB1^* | 98 | 94 | 6 | 5 | 5 | 5 | 18 |
|  | *Areg^ErbB1^* | 78 | 77 | 5 | 5 | 5 | 5 | 42 |
|  | *Btc^ErbB1/ErbB4^* | 56 | 55 | 4 | 4 | 4 | 4 | 31 |
|  | *Ereg^ErbB1/ErbB4^* | 46 | 49 | 4 | 3 | 4 | 4 | 1557 |
| **Enzymes** | *Adam17* | 793 | 826 | 7 | 7 | 7 | 7 | 165 |
|  | *Psen1* | 1353 | 1377 | 9 | 9 | 8 | 8 | 520 |
|  | *Psen2* | 172 | 168 | 6 | 5 | 6 | 6 | 32 |
|  | *Bace1* | 485 | 453 | 8 | 9 | 8 | 8 | 154 |
|  | *Bace2* | 60 | 58 | 4 | 4 | 5 | 4 | 0 |
| **Class Ia** | *Pik3ca^p110a^* | 2321 | 2355 | 9 | 10 | 10 | 9 | 234 |
|  | *Pik3cb^p110b^* | 959 | 1016 | 8 | 8 | 8 | 8 | 50 |
|  | *Pik3cd^p110d^* | 212 | 204 | 7 | 7 | 7 | 7 | 137 |
|  | *Pik3r1^p85a p55a p50a^* | 1612 | 1549 | 10 | 9 | 8 | 9 | 165 |
|  | *Pik3r2^p85b^* | 851 | 781 | 9 | 9 | 9 | 9 | 131 |
|  | *Pik3r3^p55g^* | 2496 | 2637 | 10 | 10 | 10 | 9 | 108 |
| **Ib** | *Pik3cg^p110g^* | 96 | 95 | 4 | 4 | 4 | 4 | 0 |
|  | *Pik3r5^p101^* | 96 | 97 | 5 | 5 | 5 | 5 | 31 |
|  | *Pik3r6^p87^* | 98 | 99 | 5 | 5 | 5 | 5 | 0 |
| **II** | *Pik3c2a* | 675 | 711 | 8 | 7 | 7 | 7 | 258 |
|  | *Pik3c2b* | 706 | 717 | 8 | 9 | 8 | 8 | 0 |
|  | *Pik3c2g* | 34 | 34 | 4 | 4 | 4 | 4 | 0 |
| **III** | *Pik3c3^Vps34^* | 946 | 966 | 8 | 8 | 8 | 8 | 70 |
|  | *Pik3r4^Vsp15/p150^* | 576 | 581 | 8 | 8 | 8 | 7 | 33 |
| **Controls** | *(+) Gad67* | 424 | 517 | 10 | 10 | 10 | 10 | 13 |
|  | *(+) Lhx6* | 325 | 287 | 8 | 10 | 8 | 10 | 22 |
|  | *(+) Npy* | 417 | 319 | 10 | 9 | 10 | 9 | 0 |
|  | *(+) Gapdh* | 9838 | 15302 | 12 | 12 | 12 | 12 | 5106 |
|  | *(+) Actb* | 15827 | 15863 | 12 | 12 | 12 | 12 | 5365 |
|  | *(-/+) Nkx2.1* | 71 | 79 | 5 | 9 | 5 | 7 | 0 |
|  | *(-) Pvalb* | 59 | 61 | 4 | 4 | 4 | 4 | 0 |
| **Cut-off^§^** | | **150** | | **7** | | | | **10** |

*Note that in the *p35 KO* the entire *p35* ORF and 400 bp of downstream (3’ UTR) sequence were replaced with the neo cassette in the opposite transcriptional orientation (Chae et al., 1997). However, 463 bp of upstream (5’ UTR) and last 2379 bp of downstream (3’ UTR) sequence were kept intact and, therefore, detected with the Affymetrix probes for p35 (Cdk5r1).

*Egfr*, epidermal growth factor receptor; *Erbb*, v-erb-b2 erythroblastic leukemia viral oncogene homolog; *Nrg*, neuregulin; *Hbegf*, heparin-binding EGF-like growth factor; *Tgfα*, transforming growth factor alpha; *Egf*, epidermal growth factor; *Areg*, amphiregulin; *Btc*, betacellulin; *Ereg*, epiregulin; *Adam*, a disintegrin and metallopeptidase; *Psen*, presenilin; *Bace*, beta-site APP cleaving enzyme; *Pik3*, phosphatidylinositol 3-kinase; c, catalytic; r, regulatory subunit; *Gad*, glutamic acid decarboxylase; *Lhx*, LIM homeobox; *Npy*, neuropeptide Y; *Gapdh*, glyceraldehyde-3-phosphate dehydrogenase; *Actβ*, actin, beta; *Nkx*, NK2 homeobox; *Pval*, parvalbumin.

^§^Cut-off, denoting signal threshold

**Table S3. Different interneuron migratory defects observed in *ErbB4* versus *p35* knockouts at E13.5**

|  | ***ErbB4* KO** | ***p35* KO** |
| --- | --- | --- |
| **Isoform** | Cyt2 | Cyt1 |
| **Migratory mechanism** | haptotaxis | chemotaxis |
| **Location** | MGE/LGE transition | Pallial/subpallial boundary |

**FIGURE LEGENDS**

**Figure S1. ErbBs and ErbB ligands in the developing forebrain.** (A) Schematic, illustrating *ErbB* expression in cortical cells. Solid line, expressed; dotted line, not expressed. (B) Schematic, illustrating the expression of ErbB4 ligands in the developing forebrain (this study, see Supplementary Fig. S2; Anton et al. 2004; Assimacopoulos et al. 2003; Calaora et al. 2003; Flames et al. 2004; Kornblum et al. 1999; Zhang et al. 1997). (C) Schematic, illustrating ErbB ligands, corresponding receptors and their role in chemotaxis. (D, E) Immunoblots of protein lysates from the GE and Cx of E13.5 mice, confirming presence of only one NRG1 receptor, ErbB4, but not ErbB3. Schwann cell protein lysate is used as a positive control for ErbB3 antibody. βAct serves as a loading control. (F) Chemotactic response of E13.5 MGE-derived cells to a control (white) or to an EGF-like domain of ErbB4 ligands: NRG1β (shades of green) and HB-EGF (shades of blue)]. Note a dose-dependent chemotactic response of MGE-derived cells to ErbB4 ligands reaching a plateau at 200 ng/ml (NRG1β; dark green) and 100 ng/ml (HB-EGF; dark blue). CM, conditioned medium; cp, cortical plate; Cx, cortex; GE, ganglionic eminence; M, medial; PSB, pallial-subpallial border; Str, striatum; vz, ventricular zone.

**Figure S2. NRG3 protein expression in the developing forebrain.** Neuregulin (NRG) 3 is a member of the epidermal growth factor (EGF) superfamily of membrane-bound (uncleaved) or secreted (cleaved/soluble) proteins. (A) Schematic, showing EGF-like domain (red), proteolytic cleavage region (asterisk; green), and transmembrane domain (TM; black) of NRG3. The EGF-like domain (Lys290-Asp331) contains six conserved cysteine residues (Cys1-Cys6; red). The proteolytic cleavage sites are located downstream to Phe352 and Arg361 of NRG3 close to the TM domain (Val363-Phe383). (B) Schematic, showing antibodies that recognise the EGF-like domain (Glu284-Pro344; NRG3^EGF^ antibody; red) or the cleavage region (His347-Val363; NRG3^uncleaved^; green) of NRG3. Note that NRG3^uncleaved^ antibody targets only membrane-bound NRG3 and is able to give an indication of NRG3 origin. (C) Forebrain sections of a mouse embryo, immunostained for NRG3^uncleaved^ at indicated ages. (D) Cortical sections of a mouse embryo at E15.5, immunostained for NRG3^EGF^ and NRG3^uncleaved^. (E) Cortical sections of a mouse embryo at E15.5, immunostained for NRG3^uncleaved^ and CTIP2, a marker of layer V pyramidal neurons. (F) Cortical sections of a *GAD67GFP* mouse embryo at E15.5 immunostained, for NRG3^uncleaved^. GE, ganglionic eminence; IZ, intermediate zone; LI, layer I; cp/CP, cortical plate; Cx, cortex; psb, pallial-subpallial border; SP, subplate; str, striatum; SVZ, subventricular zone.

**Figure S3. Validation for the pErbB4^T1152^ and pErbB4^Y1056^ phospho-specific antibodies.** pErbB4^T1152^ and pErbB4^Y1056^ antibodies are designed to recognise Cdk5-targeted (T1152) and PI3-kinase-binding (Y1056) phosphorylation sites, respectively. (A-B) Immunoblots of protein lysates from COS7 cells transfected with ErbB4 (first lane), ErbB4ΔICD (second lane; ICD, intracellular domain), or mutated ErbB4 (third lane), and treated with 50 ng/ml neuregulin (NRG)1β for 10 minutes; COS7 co-transfection, ErbB4 constructs with p35 and Cdk5 in (A). Lack of ErbB4 ICD or ErbB4^T1152A^ and ErbB4^Y1056F^ mutations disrupts the recognition of ErbB4 by the pErbB4^T1152^and pErbB4^Y1056^ phospho-specific antibodies, respectively. Note an incomplete abolition of pErbB4^Y1056^ immunoreactivity in COS7 cells transfected with ErbB4^Y1056F^. **(C-J) Cortical sections of wild-type (WT) GAD67^GFP^ mouse embryos at indicated ages, immunostained for phosphorylated ErbB4, pT1152 (C,G) and pY1056 (G,H); quantification of GFP^GAD67(+)^ cells that contain phosphorylated ErbB4 in the intermediate (IZ) and subventricular (SVZ) zones (E-J).** (K,L) Sections of wild-type postnatal (P21) somatosensory cortex immunostained for pErbB4^T1152^ and parvalbumin (PV). Note that pErbB4^T1152^ is present in the majority of PV^(+)^ cortical interneurons (arrow) and in some PV^(-)^ cells (asterisk). Bars, 25 μm (C,D), 75 μm (G,H), 50 μm (K,L).

**Figure S4. Validation of co-transfection efficiency in MGE cell electroporation experiments.** Focal electroporation of control (pCAG-IRES2-EGFP; referred to as pCAG; A, A’, A’’) vector and ErbB4 constructs (expressed from pCAG; B-G’’), mixed with a CAG-driven tdTomato vector, into the E13.5 medial ganglionic eminence (MGE) of the whole mouse telencephalic hemisphere. (A-G’’) Fluorescent images of GFP (green; A-G), tdTomato (red; A’-G’) or GFP/tdTomato (overlay; A’’-G’’), depicting MGE cells at the pallial-subpallial border (PSB) 48 hours after electroporation. Bar, 100 μm.

**Figure S5. Loss of *p35* or *ErbB4* does not affect mitosis or apoptosis in the forebrain.** (A-D) MGE sections of control (A and C) or *p35* (B) and *ErbB4^heart^* (D) KO mice at E13.5, immunostained for phospho histone (pH) 3, a mitotic marker. (H-I) Quantification of the number (density) of pH3 cells in the MGE of control (black) and KO (white) animals. (E-H’) Forebrain (E-H) or trigeminal ganglion (E’-H’) sections of control (E, E’, G, G’) or *p35* (F, F’) and *ErbB4^heart^* (H, H’) KO mice, immunostained for active (cleaved) caspase 3, an apoptotic marker. Bars, 50 μm (A-D), 200 μm (J-M), 50 μm (J’-M’). Cx, cortex; GE, ganglionic eminence; M, medial; L, lateral.

**Figure S6. Loss of *p35* or *ErbB4* does not affect cortical interneuron specification.** (A-C) Bar graphs, showing percentage of PV and SST cells per total GFP^GAD67^ (p35 litters; (A-B) or PV cells per total GABA (ErbB4 litters; (C) cells in the cortex of control (black) and indicated mutant (white) animals. WT, wild type; KO, knockout; Het, heterozygous; PV, parvalbumin; SST, somatostatin; ns, not significant; Student’s t-test.

**Figure S7. Both Cyt1 and Cyt2 promote chemotaxis of GN11 cells towards NRG1 in vitro; p35/Cdk5 augments this process in presence of Cyt1**

**(A) RT-PCR, revealing expression of *ErbBs, p35* and *Cdk5* in the GN11 cells. *Actβ* serves as an internal control. (B, C) Chemotactic response of FACS-purified GN11 cells 48 h after transfection with (B) control (pCAG^GFP^), Cyt1^GFP^ or Cyt1^GFP^ mixed with p35/Cdk5 or (C) control (pCAG^GFP^), Cyt2^GFP^ or Cyt2^GFP^ mixed with p35/Cdk5, to NRG1β. *p ≤ 0.05, ***p ≤ 0.005, *t*-test.**

**Figure S8. Schematics, proposing molecular (A) and cellular (B) models of cortical interneuron migration with respect to the role of p35/Cdk5 kinase and intracellular ErbB4 isoforms.** Explicitly, p35/Cdk5 kinase may regulate leading process dynamics and chemotaxis of interneurons towards secreted neuregulins (NRG) 1-Ig and 3 in the pallium (P), via interaction with the Cyt1/PI3-kinase signalling pathway. On the other hand, Cyt2-expressing interneurons may use haptotaxis in the subpallium (S), and migrate along LGE cells, replete with membrane-bound NRG1-CRD. GP, globus pallidum; LGE, lateral ganglionic eminence; MGE, medial ganglionic eminence; PM, plasma membrane; Str, striatum; TK, tyrosine kinase; vz, ventricular zone.
