# Supplementary figures and images for: Cdk5 Phosphorylation of ErbB4 is Required for Tangential Migration of Cortical Interneurons
Source: Cereb Cortex. 2013 Oct 18;25(4):991–1003. doi: 10.1093/cercor/bht290 (PMC4380000; doi:10.1093/cercor/bht290)

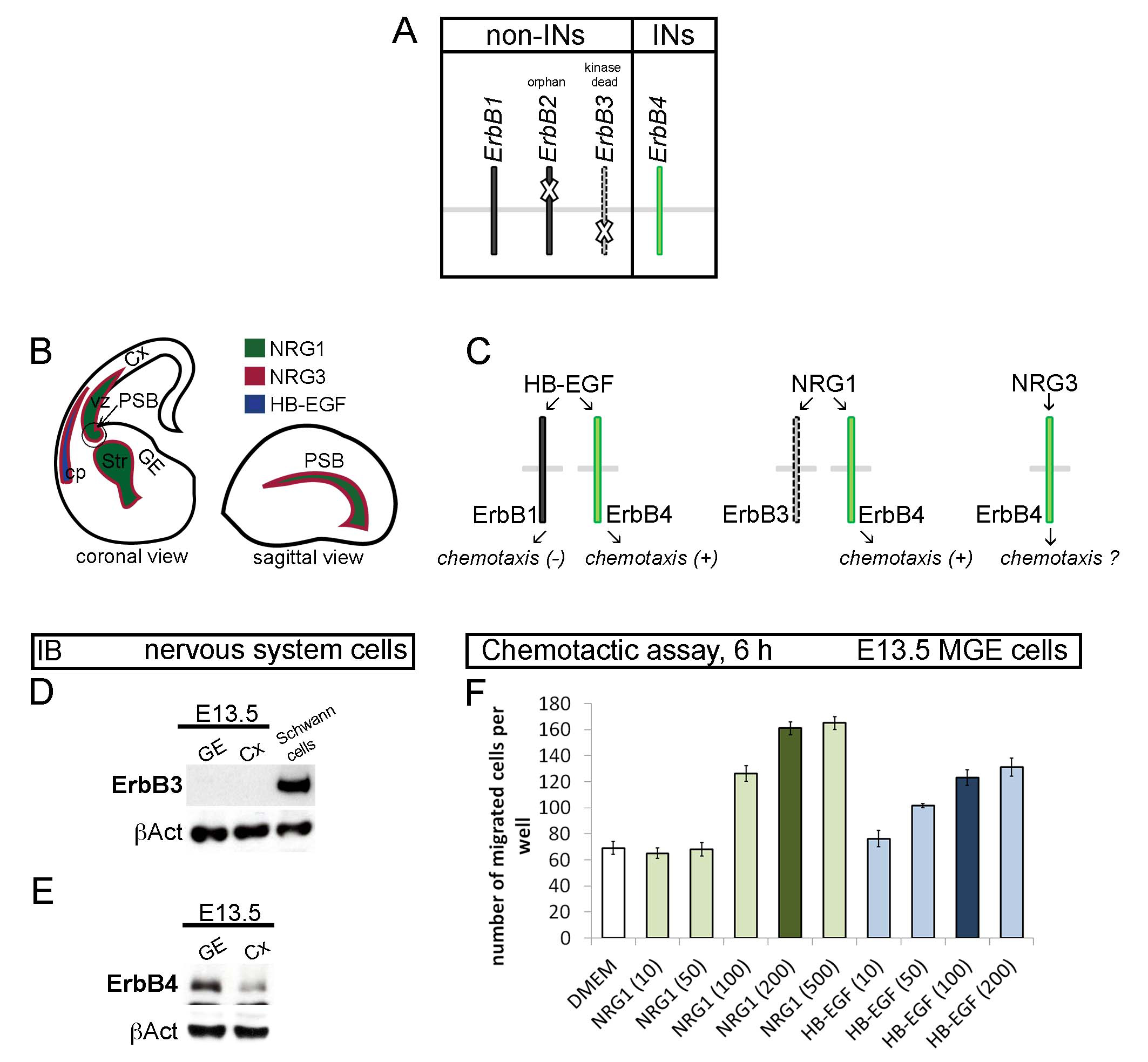

Supplement: Supplementary Data [file supp_bht290_bht290supp_fig1.jpg]

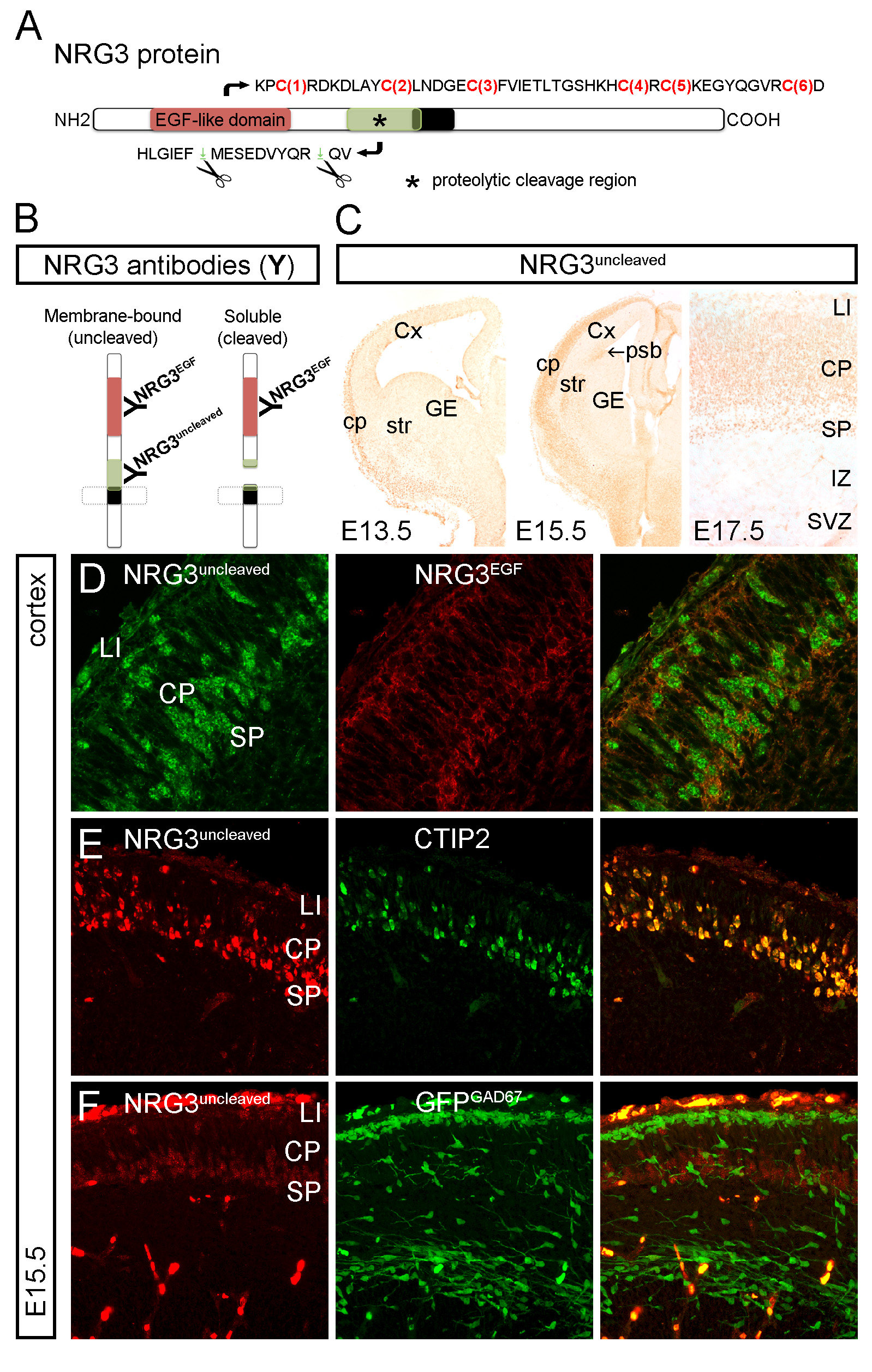

Supplement: Supplementary Data [file supp_bht290_bht290supp_fig2.jpg]

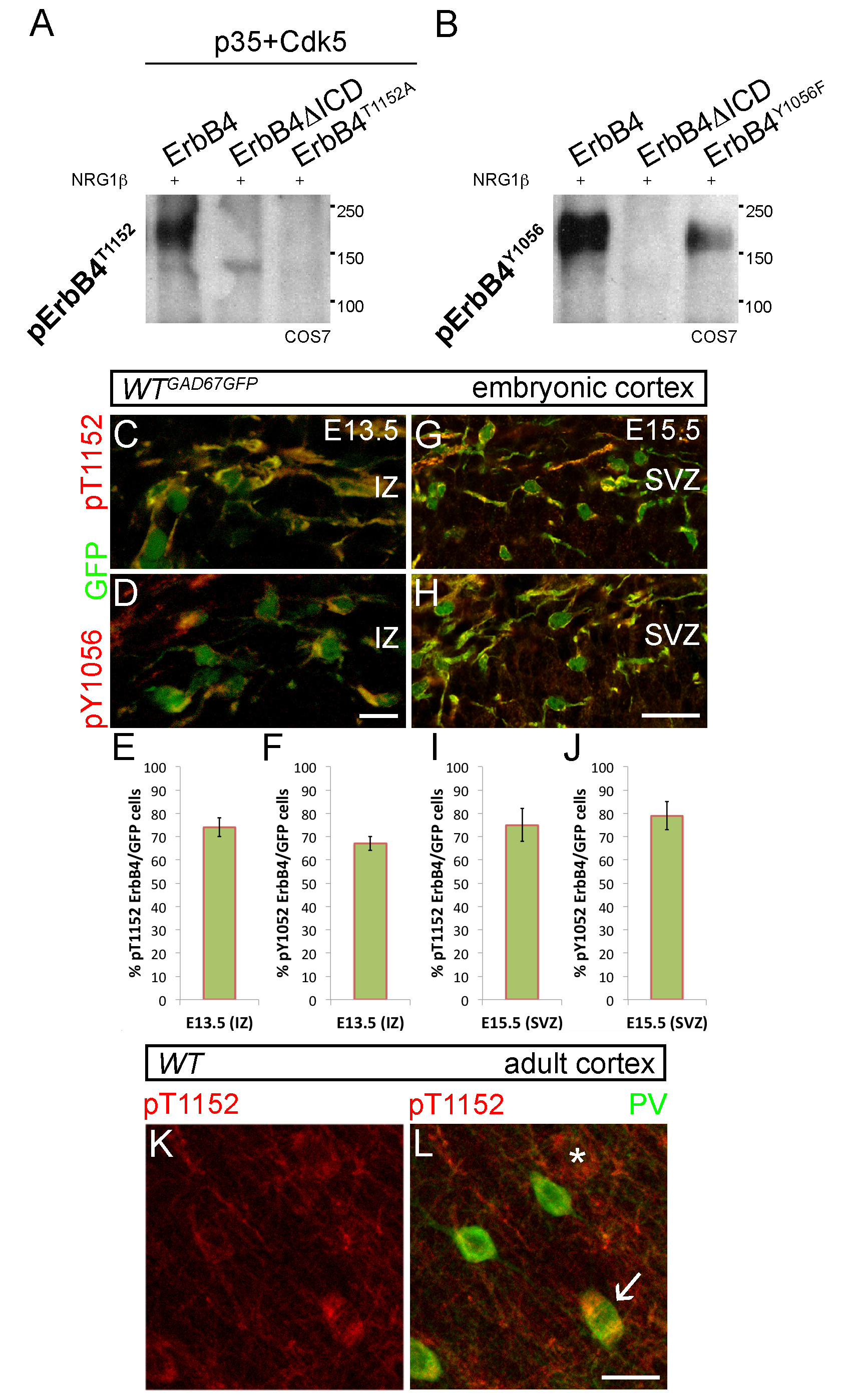

Supplement: Supplementary Data [file supp_bht290_bht290supp_fig3.jpg]

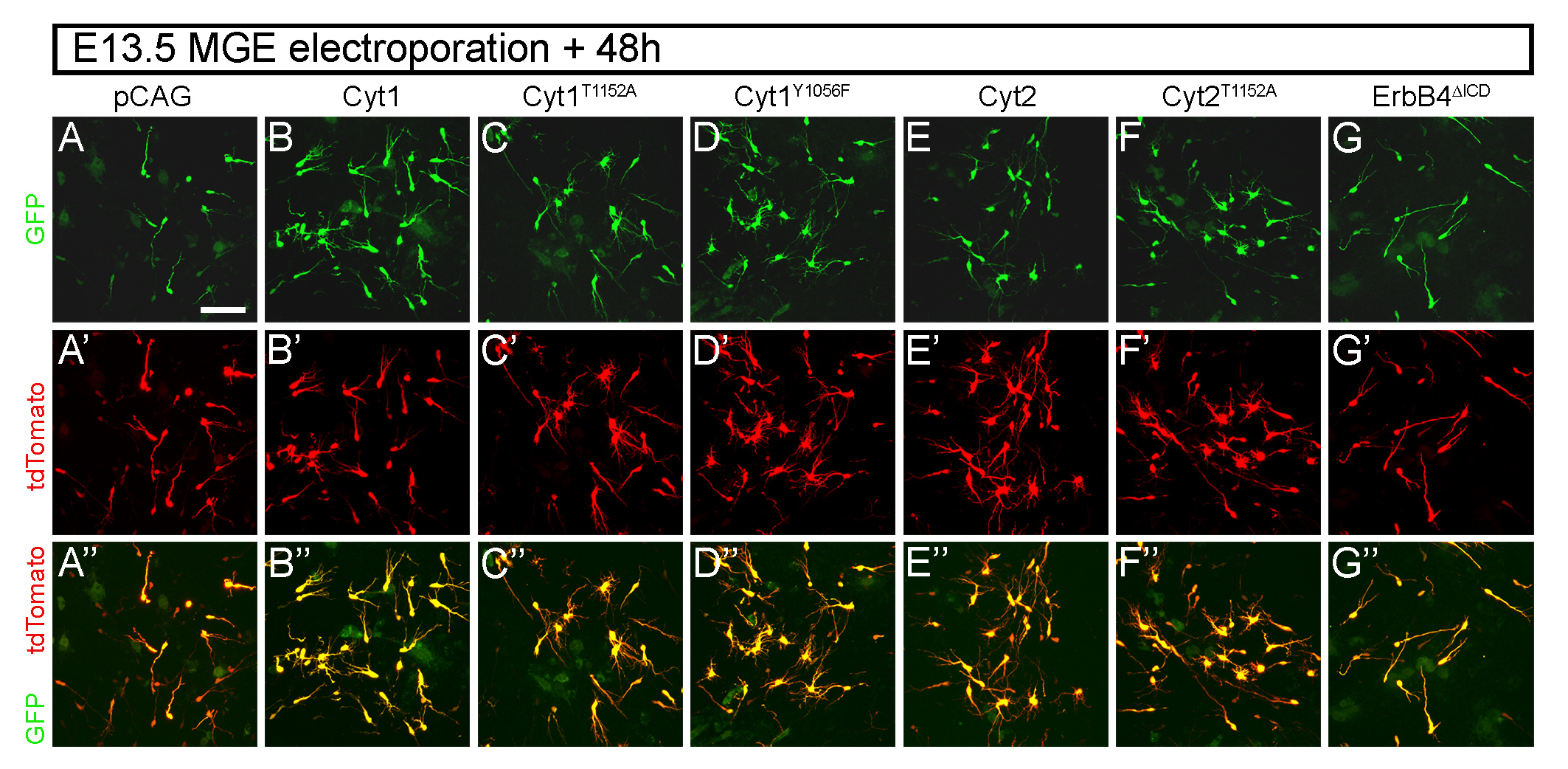

Supplement: Supplementary Data [file supp_bht290_bht290supp_fig4.jpg]

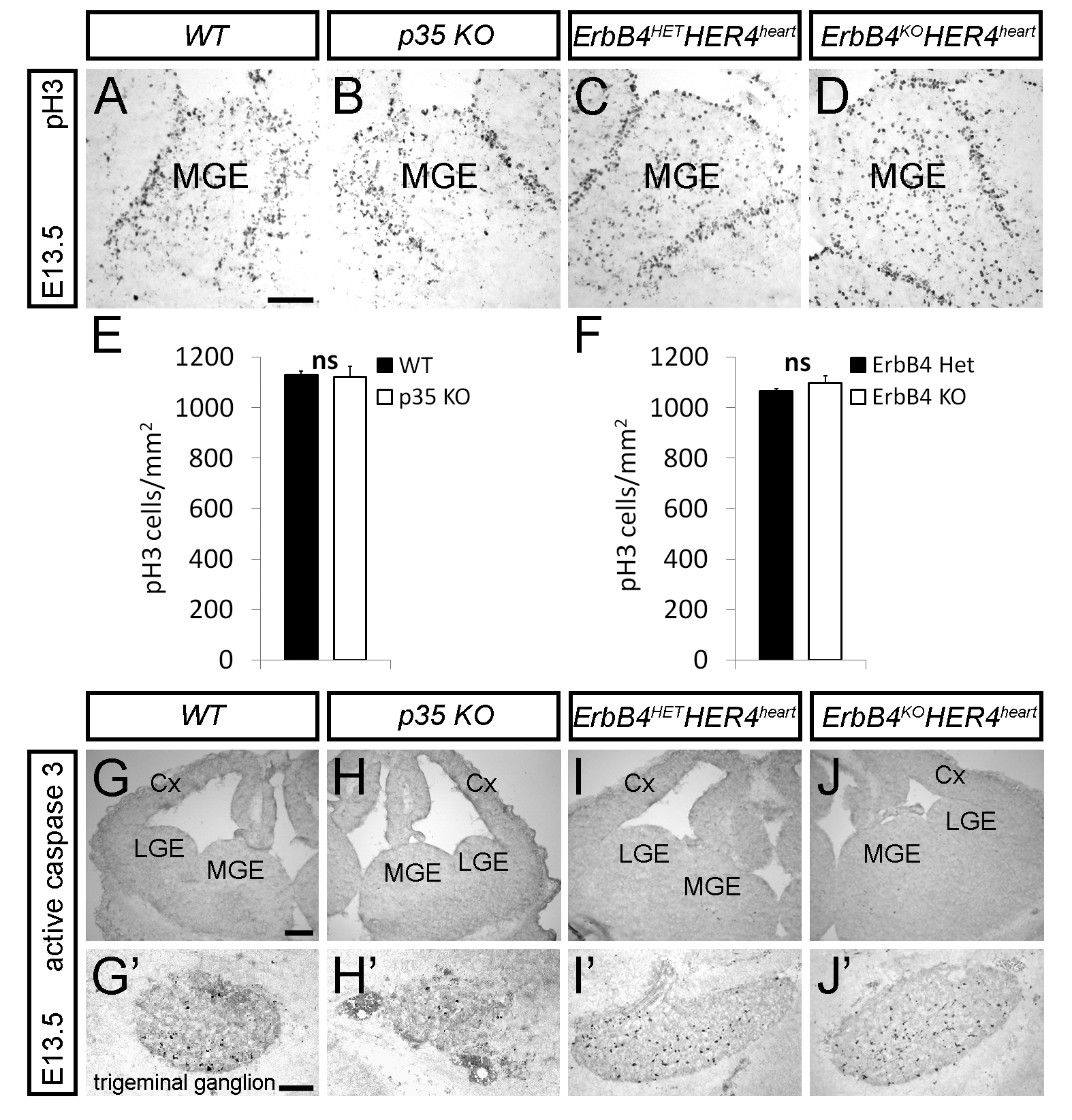

Supplement: Supplementary Data [file supp_bht290_bht290supp_fig5.jpg]

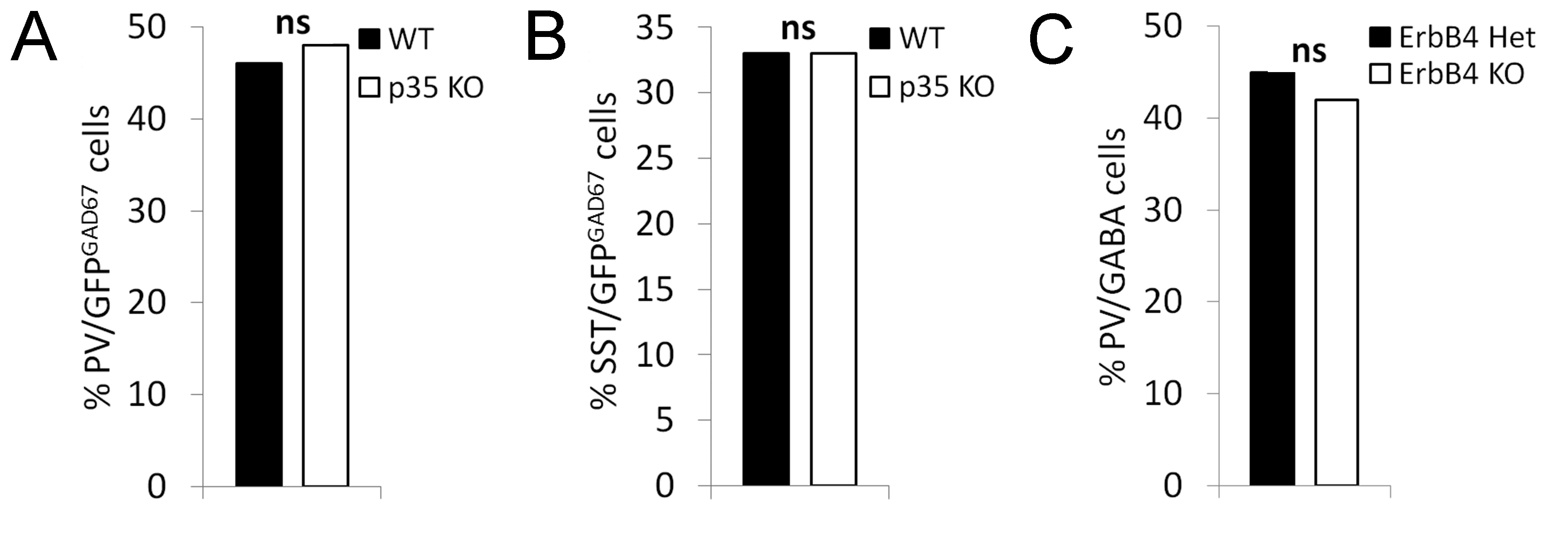

Supplement: Supplementary Data [file supp_bht290_bht290supp_fig6.jpg]

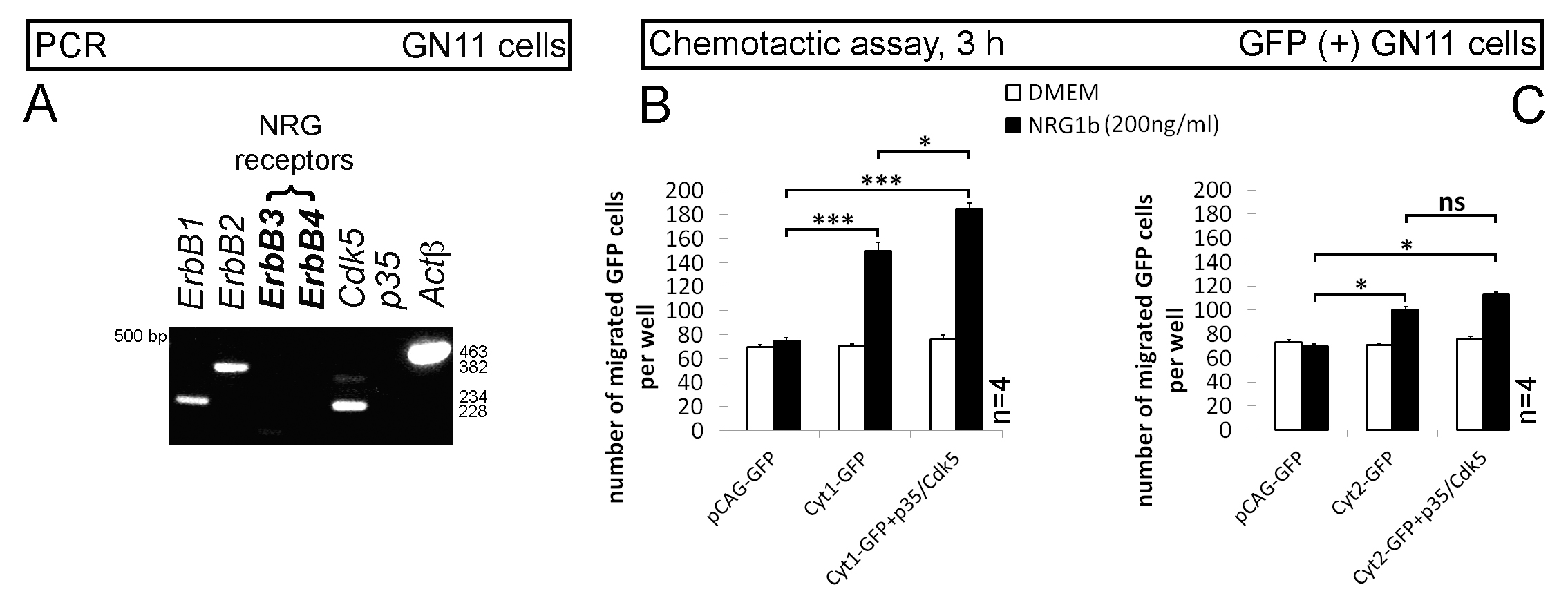

Supplement: Supplementary Data [file supp_bht290_bht290supp_fig7.jpg]

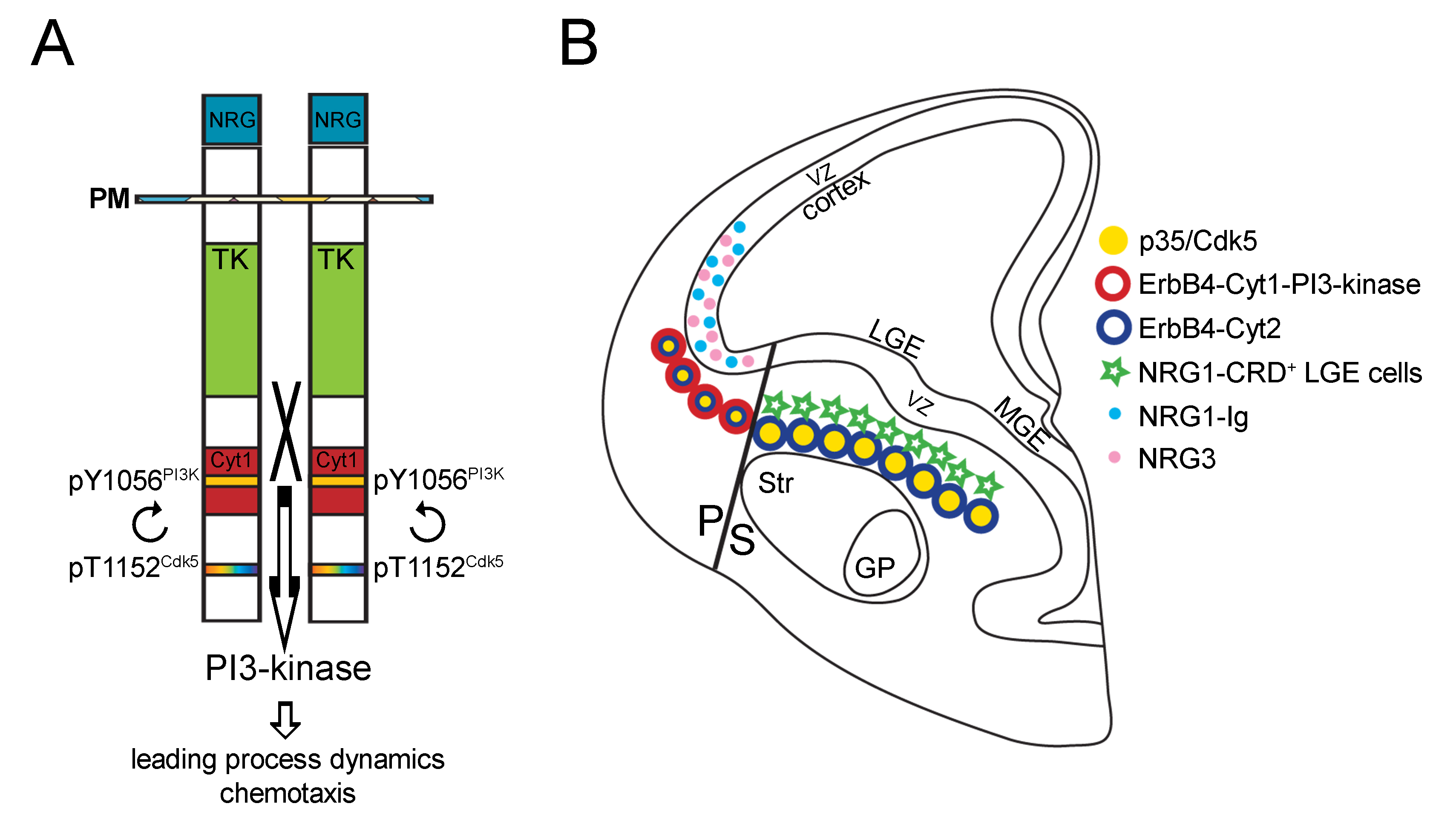

Supplement: Supplementary Data [file supp_bht290_bht290supp_fig8.jpg]
